# The implication of the air quality pattern in South Korea after the COVID-19 outbreak

**DOI:** 10.1038/s41598-020-80429-4

**Published:** 2020-12-31

**Authors:** Ja-Ho Koo, Jhoon Kim, Yun Gon Lee, Sang Seo Park, Seoyoung Lee, Heesung Chong, Yeseul Cho, Jaemin Kim, Kyungbae Choi, Taegyung Lee

**Affiliations:** 1 Department of Atmospheric Sciences, Yonsei University, Seoul, Republic of Korea; 2 Department of Atmospheric Sciences, Chungnam National University, Daejeon, Republic of Korea; 3 School of Urban and Environmental Engineering, Ulsan National Institute of Science and Technology, Ulsan, Republic of Korea

**Keywords:** Environmental sciences, Environmental social sciences

## Abstract

By using multiple satellite measurements, the changes of the aerosol optical depth (AOD) and nitrogen dioxide (NO_2_) over South Korea were investigated from January to March 2020 to evaluate the COVID-19 effect on the regional air quality. The NO_2_ decrease in South Korea was found but not significant, which indicates the effects of spontaneous social distancing under the maintenance of ordinary life. The AODs in 2020 were normally high in January, but they became lower starting from February. Since the atmosphere over Eastern Asia was unusually stagnant in January and February 2020, the AOD decrease in February 2020 clearly reveals the positive effect of the COVID-19. Considering the insignificant NO_2_ decrease in South Korea and the relatively long lifetime of aerosols, the AOD decrease in South Korea may be more attributed to the improvement of the air quality in neighboring countries. In March, regional atmosphere became well mixed and ventilated over South Korea, contributing to large enhancement of air quality. While the social activity was reduced after the COVID-19 outbreak, the regional meteorology should be also examined significantly to avoid the biased evaluation of the social impact on the change of the regional air quality.

## Introduction

The severity of social problems due to air pollution (e.g., health problems, agricultural damage, economic effect) is widely known to the point where many people are concerned about their life quality^1–4^. Particularly, East Asia shows the most serious air pollution in the world, and it needs urgent countermeasures for enhancing its regional air quality^5^. Recently, some improvements of the air quality in China and Korea have been reported^6,7^. However, these enhancements are still not enough to solve the regional air pollution problems. The difficulty in finding a solution basically comes from the complexity of atmospheric chemistry, which is still not clearly understood. Also, several recent studies have revealed that serious air pollution issues not only result from the local emissions, but they also take place due to the influence of the regional meteorology and large-scale circulation. For example, the high concentration of air pollutants in East Asia tends to be associated with the low wind speed^8^, the weakened Siberian High pressure^9,10^, the occurrence of eastern Pacific El-Niño^11^, and even the decline in the Arctic sea ice^12,13^. These studies have raised suspicions regarding human efforts when it comes to finding a solution for air quality problems.

Under these circumstances, we suddenly confronted a dreadful accident damaging to the whole global society, coronavirus disease 2019 (COVID-19). After the COVID-19 outbreak, global death toll has reached more than one million, and the number of confirmed cases has become more than 38 million (as of October 2020). Due to this outrageous damage, many social activities have been restricted to stop the spread of the disease^14^. In spite of this negative face, people have also tried to find a positive merit associated with the COVID-19 so as to advance our society in the future^15^. In particular, people now focus on the emission decrease of the anthropogenic air pollutants driven by social distancing and the contracted economic activity, resulted in the improvement of regional air quality^14^. Since the possibility of an interactive feedback process between the local air pollution and mortality has been reported^16^, the relationship between the air quality and social activity is receiving more attention^17^.

Several works have already reported diagnoses and analyses regarding the changes in the regional air quality under the COVID-19 epidemic. Surface in-situ measurements generally detected a huge decrease of the air pollutants after the COVID-19 outbreak^18,19^, and satellite measurements revealed a huge reduction of the high-emission areas^20,21^. However, the surface ozone level was often enhanced, which was interpreted as a result of the lower NO_x_ emissions^19,22^. These urgent inspections are useful for seeing what is happening now related to the COVID-19 spread, but several evaluations look quantitatively uncertain because the analyses targeted a very short-time variation after the COVID-19 outbreak or a simple comparison to the previous year (2019)^18^. Also, most of these quick reports included a simple interpretation of the observed data from the viewpoint of the emission change^21^, which can overlook other influential factors associated with the air pollution, such as the meteorology effect. Since some studies have indicated the peculiar patterns of the airborne aerosol concentration during the lockdown periods related to the regional meteorology pattern^23–25^, careful analyses are further required for more accurate conclusions.

This study investigated the air quality change before and after the COVID-19 outbreak in comparison with the recent 5-year mean pattern of air pollution. Also, we put more weight on the regional meteorology analysis so as to better evaluate the extent of the emission-down effect. The previous studies dominantly treated cases in China where the most severe air pollution occurs in the world. Here, we examined the situation in South Korea. The case in South Korea has a similar context to previous works about the COVID-19 effect to the air pollution in China^21,24^, but we expected to acquire new patterns based on this research because of the different situation in South Korea. Despite the huge damages related to the COVID-19, South Korea did not execute city lockdowns or any intensive control strategies of social activities^26^. At most, a number of companies conducted telecommuting, schools provided online lectures, and private activities were spontaneously regulated. As a result, the distribution system of commercial products was almost maintained without serious business shutdowns or economic problems. Thus, the air quality pattern in this situation may be different from that in other countries. Overall, this study can reveal interesting findings for the research community of atmospheric environments.

## Results

In South Korea, a rapid increase of the COVID-19 confirmed cases occurred in late February 2020 in Daegu, a metropolitan city located in the southeastern part of the Korean peninsula, and it took place due to closed congregation gatherings. This trend continued and then resulted in a large number of confirmed cases > 10 thousands in late March (Fig. S1). Thus, we investigated the relationship of air quality to the COVID-19 in South Korea from January to March 2020 in terms of three phases: January (as a normal condition), February (when the COVID-19 spread happened), and March (when the social activity was shrunk to avoid the COVID-19 spread). This monthly analysis in 2020 was compared to the pattern in the same months of 2019 and also to the mean monthly pattern during the recent 5 years (2016–2020). Spatially, we focused on two regions as representative examples: Seoul and the Gyeonggi province (SG), which is in the center of South Korea, and Daegu and the Gyeongbuk province (DG), which had the biggest number of COVID-19 confirmed cases during the research time period. Since the air quality in the Korean peninsula is strongly affected by the transboundary transport of air pollutants from China^27,28^, we also simultaneously investigated the air quality pattern in 4 provinces of eastern China (Liaoning, Hebei, Shandong, and Jiangsu) (Fig. S2). To cover this wide area, we mainly used satellite measurements for the aerosol optical depth (AOD) and the nitrogen dioxide (NO_2_) vertical column density (VCD).

First, we examined the monthly mean patterns of the AOD and NO_2_ VCD in East Asia. For the AOD analysis, we used the AOD at 550 nm from the measurements of the Geostationary Ocean Color Imager (GOCI) onboard the Communication, Ocean and Meteorological Satellite (COMS). The January mean AODs in eastern China and in the west of the Korean peninsula were generally higher in 2020 in comparison with the pattern in 2019 and with the average pattern in 2016–2020. However, lower AODs were partially found during February and March in comparison with the cases in 2019 and in 2016–2020 (Fig. S3). While AODs are still high around Beijing, a decline in the February and March AODs is obvious in southeastern China (Figs S4 and S5), which seems to reflect the emission decrease resulted from the lockdown of Wuhan in late January 2020. Thus, it is expected to see weaker transboundary transport of air pollutants. In fact, the Yellow Sea and the west of the Korean peninsula, which are the downwind regions of the typical westerly in the northern hemispheric mid-latitude, have had a large AOD decrease in February and March 2020.

In a similar manner, the satellite measurements of the NO_2_ VCD were investigated. The tropospheric monitoring instrument (TROPOMI) measurement has a better spatial resolution, but it has a temporal shortage, not providing any information before 2018. Therefore, we mainly analyzed the NO_2_ VCDs from the ozone monitoring instrument (OMI) measurement having longer monitoring history, and compared with results using the TROPOMI data complementally. Their spatial distribution was quite consistent despite a little overestimation of the OMI NO_2_ VCDs (Fig. S6 and S7). Both the OMI and TROPOMI measurement indicated lower NO_2_ VCD in 2020 than 2019, and also the OMI measurement showed lower NO_2_ VCD in 2020 compared with the average pattern in 2016–2020. This reduction in 2020 seemed to be the largest in Southeastern China (Figs. S8, S9, S10, and S11). However, the monthly variation was slightly different from the AOD pattern; The decrease of the NO_2_ VCD was the strongest in January (Figs. S10 and S11), whereas the AOD decrease was more obvious in February and March (Fig. S5). This may be attributed to the lifetime difference. Namely, the NO_2_ directly reflected the lockdown effect on time due to its short lifetime, but the AOD showed a slower change as the aerosol has a relatively long lifetime^29^.

We also compared the monthly mean of the AOD and NO_2_ VCDs at our target regions, SG and DG. Between SG and DG, the AOD values were comparable (Fig. S12), but the NO_2_ VCDs were significantly different (Figs. S13 and S14). This pattern means that the NO_2_ concentration better describes the difference of air quality in a small scale, mainly attributed to the different anthropogenic activity, as the population at SG is > 20 million, but that of DG is ~ 5 million. In contrast, the AOD pattern was also affected by large scale factors, such as the mentioned transboundary transport or the influence of synoptic atmospheric circulation. Therefore, more careful analysis is required for examining the spatiotemporal pattern of AOD.

**Figure 1:**
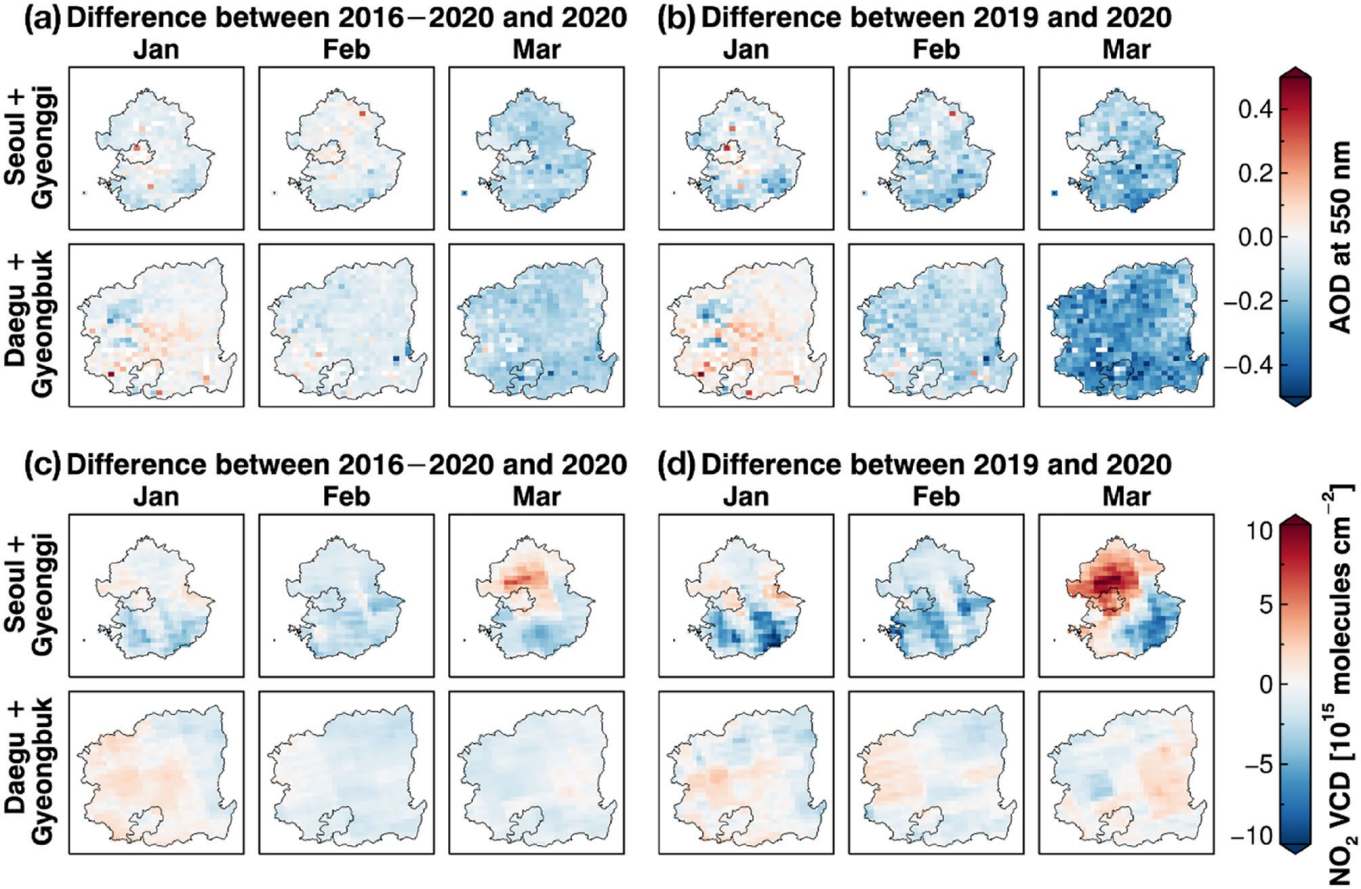
Changes in the January, February, and March means of the GOCI AOD at 550 nm in 2020 over Seoul and Gyeonggi province and Daegu and Gyeongbuk province in South Korea, comparing with those (**a**) in recent 5 years (2016–2020) and (**b**) in 2019. (**c**,**d**) are same with (**a**,**b**) but for the OMI NO_2_ VCD. The blue color scale indicates the lower AOD/NO_2_ VCD values in 2020, and the red color scale indicates the higher AOD/NO_2_ VCD values in 2020. The district geometries in South Korea are available from http://www.gisdeveloper.co.kr/?p=2332. Figures generated with Interactive Data Language (IDL) version 8.8.0 (https://www.l3harrisgeospatial.com/Software-Technology/IDL).

To investigate the South Korean situation in earnest, we compared the differences in the monthly means of the AOD and NO_2_ VCDs between 2020 and 2019 (i.e., 2020 minus 2019) and between 2020 and 2016–2020 (i.e., 2020 minus 2016–2020) in SG and DG (Fig. 1). Both the AOD and NO_2_ VCDs in 2020 were generally lower than those in 2019 and 2016–2020, but they were not drastic. In January, monthly mean AODs and NO_2_ VCDs in 2020 were not lower in both SG and DG compared with the previous years (even higher). However, the AODs and NO_2_ VCDs in 2020 were lower in February than those in other years. In March, the decline of AODs in 2020 became more significant in SG and DG as expected. However, the NO_2_ VCDs in March 2020 was not clearly lower, even higher over some areas of SG (Fig. 1d). This rebound of NO_2_ VCDs in March, which is consistently found in the analysis of TROPOMI data (Fig. S15), looks weird because the emission decrease is supposed in March 2020 when the COVID-19 confirmed cases rapidly increased. If the local emission change is the dominant factor affecting the air quality pattern, at least the NO_2_ VCDs in 2020 should be much lower than those in the other years because the AOD pattern cannot be explained only by the local emission change; As mentioned above, the influence of the external factors should be considered simultaneously for the AOD analysis. Here we presume that the air quality improvement in South Korea occurred during the COVID-19 spread, but it was not very significant.

**Figure 2:**
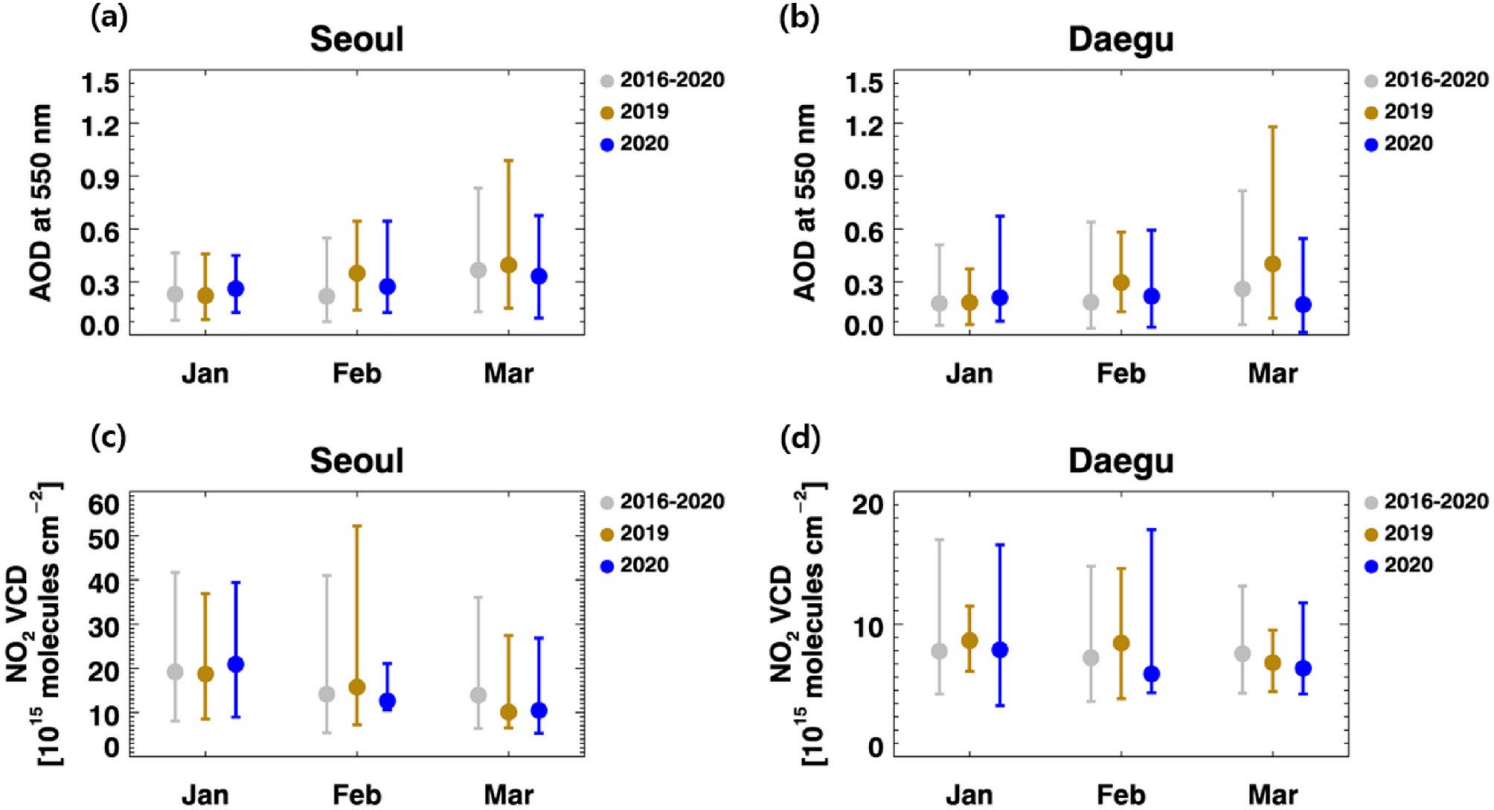
Percentiles of the observed surfaces of the GOCI AOD at 550 nm in (**a**) Seoul and (**b**) Daegu, and OMI NO_2_ VCD in (**c**) Seoul and (**d**) Daegu, South Korea for January, February, and March. (The 10th, 50th, and 90th percentiles of January, February, and March are compared for 3 periods: 2016–2020 (gray), 2019 (yellow), and 2020 (blue).

Specifically, we compared the monthly 10th, 50th (median), and 90th percentiles of the AODs and NO_2_ VCDs in Seoul and Daegu for 3 periods: 2020, 2019, and 2016–2020 (Fig. 2). Similar to Fig. 1, the median values of the AOD and NO_2_ VCDs in 2020 were higher than in the previous years in January, but they became lower in February and March in both regions. The decline of AOD and NO_2_ VCDs in February and March 2020 were obvious compared with patterns in 2019: 22% and 26% decrease of AOD, and 20% and 27% decrease of NO_2_ VCDs in Seoul and Daegu during February 2020. But decline pattern is unclear if compared with the 5-year mean pattern in 2016–2020: 25% and 18% increase of AOD, and 11% and 16% decrease of NO_2_ VCDs in Seoul and Daegu during February 2020. Since the AODs and NO_2_ VCDs in 2019 were relatively high in recent five years, the comparison of the air pollution between 2020 and 2019 may overestimate the air quality improvement. Thus, we need to carefully consider the previous evaluation of the COVID-19 effect on the local air quality if it focuses on the short-term change between 2020 and 2019. The decrease of NO_2_ VCDs in February and March 2020 looks consistent in both Seoul and Daegu, which means that the COVID-19 outbreak influenced the decrease of the anthropogenic emissions. However, again, the improvement does not look much dramatic compared with the mean pattern of previous 5 years.

**Figure 3:**
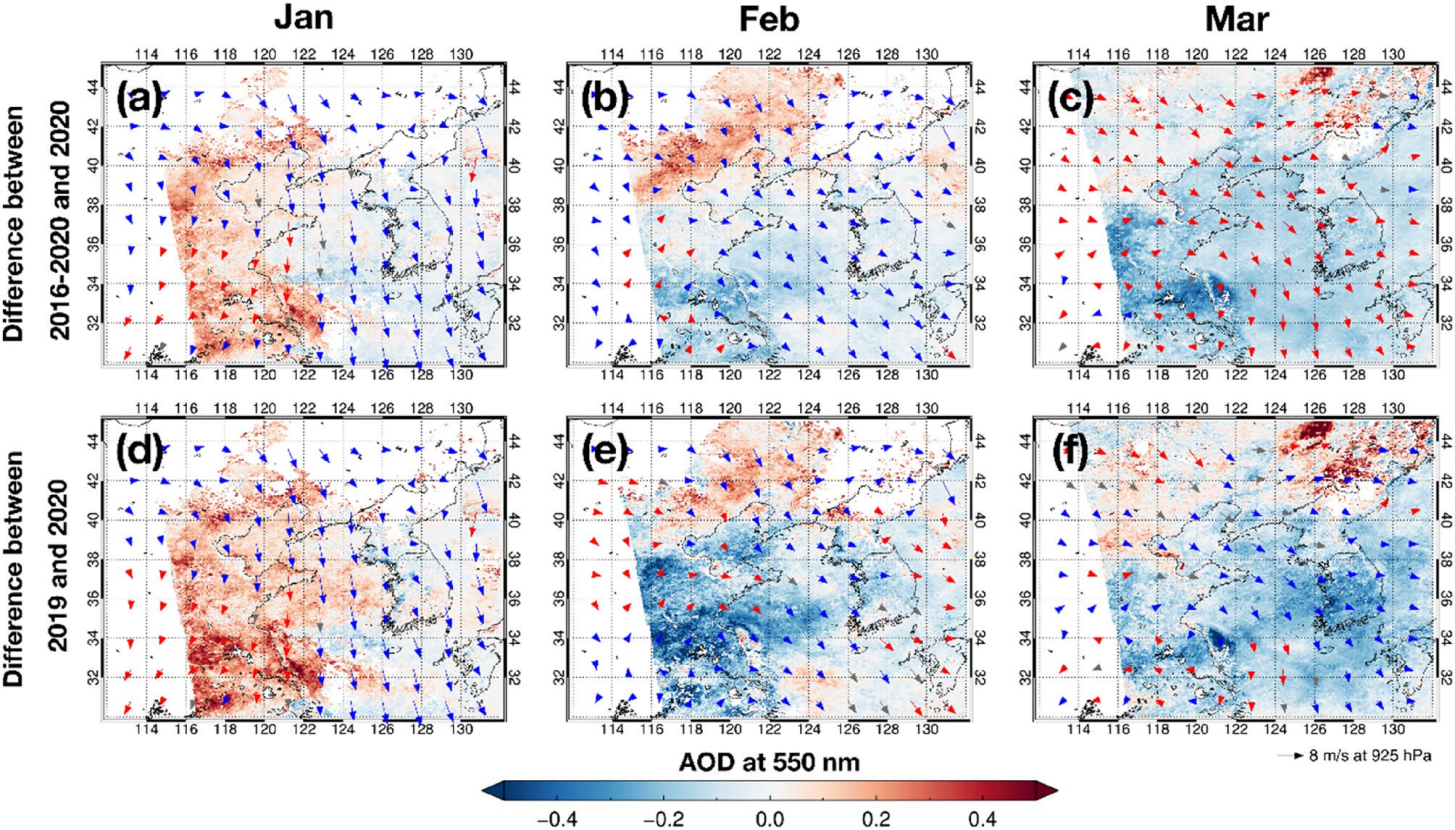
Differences in the (**a**) January, (**b**) February, and (**c**) March means of the GOCI AOD at 550 nm and the ERA5 wind field between 2020 and 2016–2020. (**d**–**f**) are same but between 2020 and 2019. The Blue color indicates the lower AOD and slower wind speed in 2020, and the red color indicates the higher AOD and faster wind speed in 2020. Figures generated with Interactive Data Language (IDL) version 8.8.0 (https://www.l3harrisgeospatial.com/Software-Technology/IDL).

Owing to the relatively long lifetime of airborne aerosols as menitoned, the variation in the AOD may not result from the change of the anthropogenic emissions. Among other possible factors, the regional meteorology pattern can dominantly induce a spatiotemporal variation of the AOD, which is largely associated with the intensification of the transboundary transport or to the stagnant air condition. Thus, we also need to examine how the meteorological pattern relates to the variation in the AOD around the Korean peninsula. For this purpose, we connected the wind field to the AOD distribution in East Asia, showing the large influence of the strong northwesterly (Fig. S16). This wind pattern is induced by the intensified Siberian high pressure, called as the East Asian winter monsoon^10^. Figure 3 shows the difference of the monthly mean AOD and wind pattern (wind speed and direction) between 2020 and 2019 (i.e., 2020 minus 2019) and between 2020 and 2016–2020 (i.e., 2020 minus 2016–2020). Interestingly, January and February show that the wind speed in 2020 were the lowest in recent 5 years over the Yellow Sea and the Korean peninsula. Considering that previous haze and smog events in eastern China and South Korea usually occurred when the regional wind was strongly weakened^10,12^, this feature reveals that strong air pollution could have taken place in January and February 2020 due to the stagnant air condition.

Since the high AOD enhancement in January 2020 can be confirmed over the whole of East Asia, particularly in eastern China (Fig. 3), we anticipated that the mean AOD in February over East Asia should be higher than in the previous years related to the low wind speed. However, the satellite measurements show different features from our expectations; they show that the AODs in February 2020 were lower than those in the previous years over most of eastern China, the Yellow Sea, and the Korean peninsula (Fig. 3). This lower AOD in February can be seen due to the effect of anthropogenic emission decrease, which is related to the lockdowns. However, the reverse pattern also occurred partially in eastern China located > 40° N: a large AOD enhancement in February 2020, corresponding to the expected pattern under the stagnant air condition. This north–south contrast of the aerosol pollution in eastern China was discussed^19^, indicating that the existence of unexpected high aerosol concentration in the Jing-Jin-Ji area resulted from the intensified secondary aerosol formation, under the highly humid and stagnant air conditions during the lockdown period along with some uninterrupted emissions. Anyway, the emission decrease in central and southern China was drastic, which seems accompanied with the diminished AOD in the Korean peninsula despite the weakened wind speed. This pattern shows that the reduction of anthropogenic emission is the basic approach for the enhanced air quality.

We find that the March cases require further consideration. The mean wind speed in March did not differ between 2020 and 2019. However, compared with the 5-year mean pattern in 2016–2020, the mean wind speed in March in East Asia was much higher in 2020 (Figs. 3 and S16). Namely, the air condition in March 2020 was not stagnant any more, but it was well ventilated, inducing the lower air pollution. Thus, it is difficult to judge whether the enhanced air quality in March was attributed to the local emission decrease or to the meteorology effect. Since we already confirmed the effect of the emission decrease in February, we may reach a similar conclusion: possibility of low AODs in March resulting from the continuous emission control after the COVID-19 outbreak. Considering that the city lockdown was a temporary action, and the emission level was actually back soon after, as confirmed by situations in China^21^, however, we cannot rule out the scenario that cleaner air quality in South Korea in March could be maintained due to the strong air ventillation while the anthropogenic emissions returned to their original levels. In other words, the analysis without the meteorological analysis in South Korea^30^ may include the overestimation of social-distancing effect for March 2020. Additional studies based on the correct emission estimation let us have a better diagnosis later.

**Figure 4:**
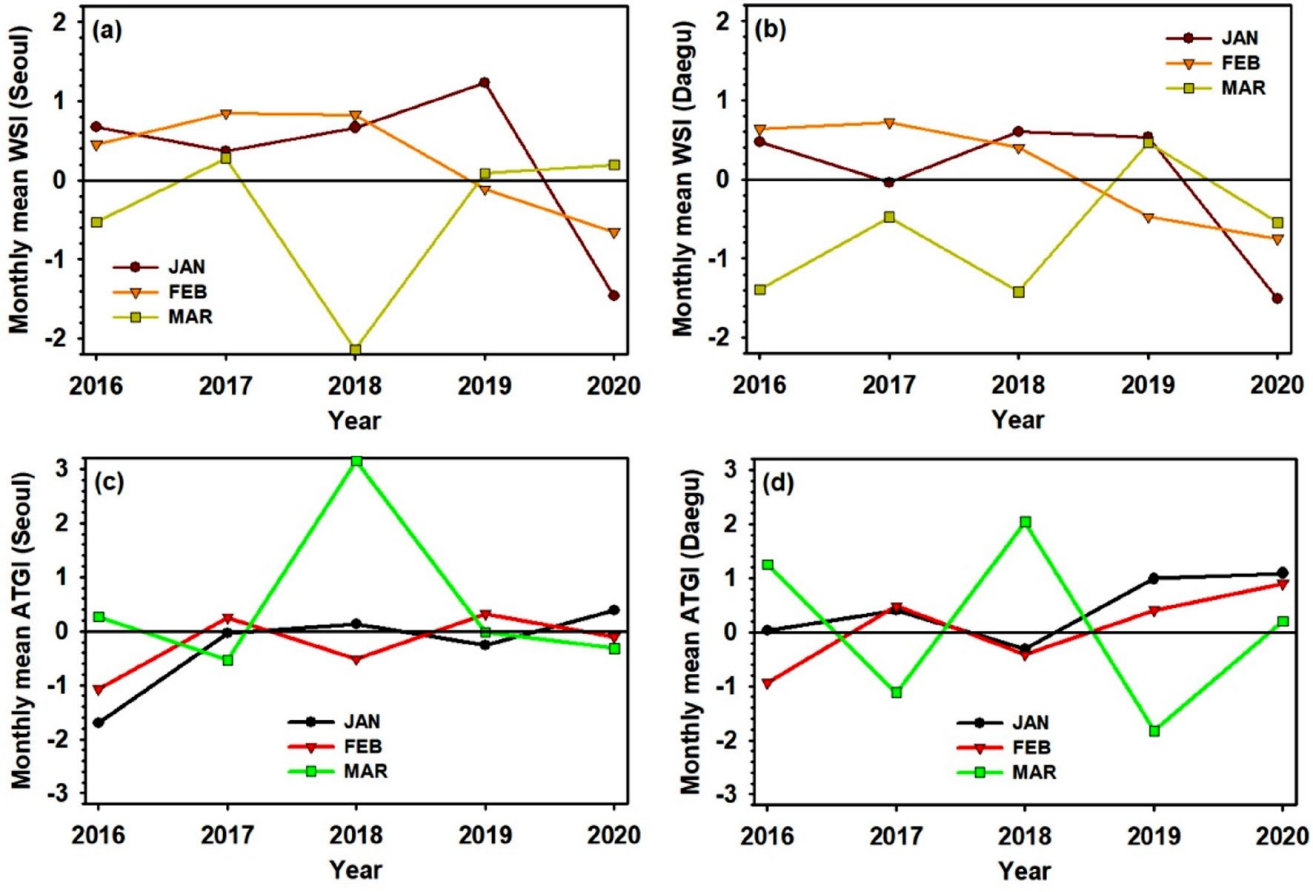
January, February, and March mean wind speed index (WSI) in (**a**) Seoul and (**b**) Daegu, and air temperature gradient index (ATGI) in (**c**) Seoul and (**d**) Daegu from 2016 to 2020.

We more performed the detail evaluation of the regional meteorology effect. Here, we estimated two additional known indices: the wind speed index (WSI) and the air temperature gradient index (ATGI)^11^ for January, February, and March from 2016 to 2020. The spatial distribution of the WSI (Fig. S17) illustrated that the January and February mean wind speed over the whole of East Asia was strongly weakened in 2020. Additionally, the spatial distribution of the ATGI (Fig. S18) depicts that the vertical air stability over the Korean peninsula and the Yellow Sea was prominently strengthened in January and February 2020. However, the WSI and ATGI patterns in March were not special in comparison with the other years. When we focused more on the 5-year variation of the WSI and ATGI in Seoul and Daegu (Fig. 4), both regions also showed the lowest WSI and highest ATGI in January and February 2020, which is in contrast to the normal situation as happened in March 2020. In brief, the East Asian air condition in January and February 2020 was favorable to have a severe accumulation of air pollutants if the common emission level was retained. The air quality in January 2020 was poor as supposed (Fig. 2). However, owing to the remarkable decrease of local emissions after the COVID-19 outbreak, we could avoid the serious deterioration of air quality in February.

## Discussions

First, the diagnosis of air quality change can be different according to the target air pollutants. This sounds hackneyed, but a number of previous works did not often consider this fact^18,30^. In terms of the COVID-19 effect, we found that the analysis of the changes in NO_2_ emissions was a standard approach as several recent studies conducted^20,21^ because of its short lifetime in air. However, this kind of trace gas is invisible, therefore people do not directly feel its variation. In contrast, the variation of the aerosol concentration can obviously be witnessed. Since the air quality change after the COVID-19 outbreak was a social spotlight issue, the analysis of the aerosol pattern better matched to the experiences of people. The problem is that the spatiotemporal pattern of the AOD and NO_2_ VCD is not clearly same as shown in our analysis. Since the amount of aerosols is also attributed to natural aerosols (e.g., mineral dust and sea salt), we also need to carefully investigate the aftereffect of the changes in the anthropogenic aerosol emissions. If this analysis extends to the springtime, we should remove the effect of Asian dust. Additionally, the regional meteorology should be considered for the accurate examination of the aerosol patterns, as conducted in this study.

Careful consideration for the data difference is also important in the comparison of same data from different sampling. For instance, AOD analysis using observations of moderate resolution imaging spectroradiometer (MODIS) indicated the broader region of high AOD in China after the COVID-19 outbreak^23^, which is different from our findings (Fig. S5). This difference can come from the sampling difference because the MODIS (onboard polar-orbit satellites) only performs a daily measurement only once in the mid-latitude region. Thus, the result using the MODIS AOD may show the much limited situation compared to results from multiple daily measurements of the GOCI (~ 8 times per day).

Second, we need to verify that our finding is valid when surface in-situ measurements are applied. Thus, we investigated the mass density of particulate matter for a diameter < 2.5 µm (PM_2.5_) and NO_2_ mixing ratios in Seoul and Daegu (Fig. S19). In general, both the PM_2.5_ and NO_2_ in 2020 maintained the mean pattern in January, but they decreased in February and March a little, which is similar to the pattern of the AOD and NO_2_ VCD (Fig. 2). But some differences were found. For example, the decline of NO_2_ in February and March 2020 is less prominent that results based on satellite data: only 2–7% decrease in Seoul and Daegu compared to the mean NO_2_ level in 2016–2020. Also in March 2019, lower NO_2_ VCD and higher surface NO_2_ patterns were observed in comparison with the previous years. This disagreement can be generated from the difference of the horizontal resolution, sampling time, and concentration between the surface and column observations. These features reveal that the analysis can reach to different conclusions according to the physical meaning of treated parameters. Thus, it is required to examine multiple dataset for more accurate analysis.

Third, we found that the short-term analysis may not be enough for evaluating the change in air quality. The AODs and NO_2_ VCDs in 2020 were lower in comparison with 2019, but they were analogous compared with the recent 5-year average, as shown in Fig. 2. The recent works on the COVID-19 effect were mostly based on comparisons with 2019. Even several studies performed simple comparisons among classified periods according to the number of COVID-19 confirmed cases or before and after the city lockdowns. These approaches were usually urgent reports, therefore we appreciate their contribution of useful information to the research community at this time. Nonetheless, future studies need to use long-term data as much as possible to improve our understanding.

**Figure 5:**
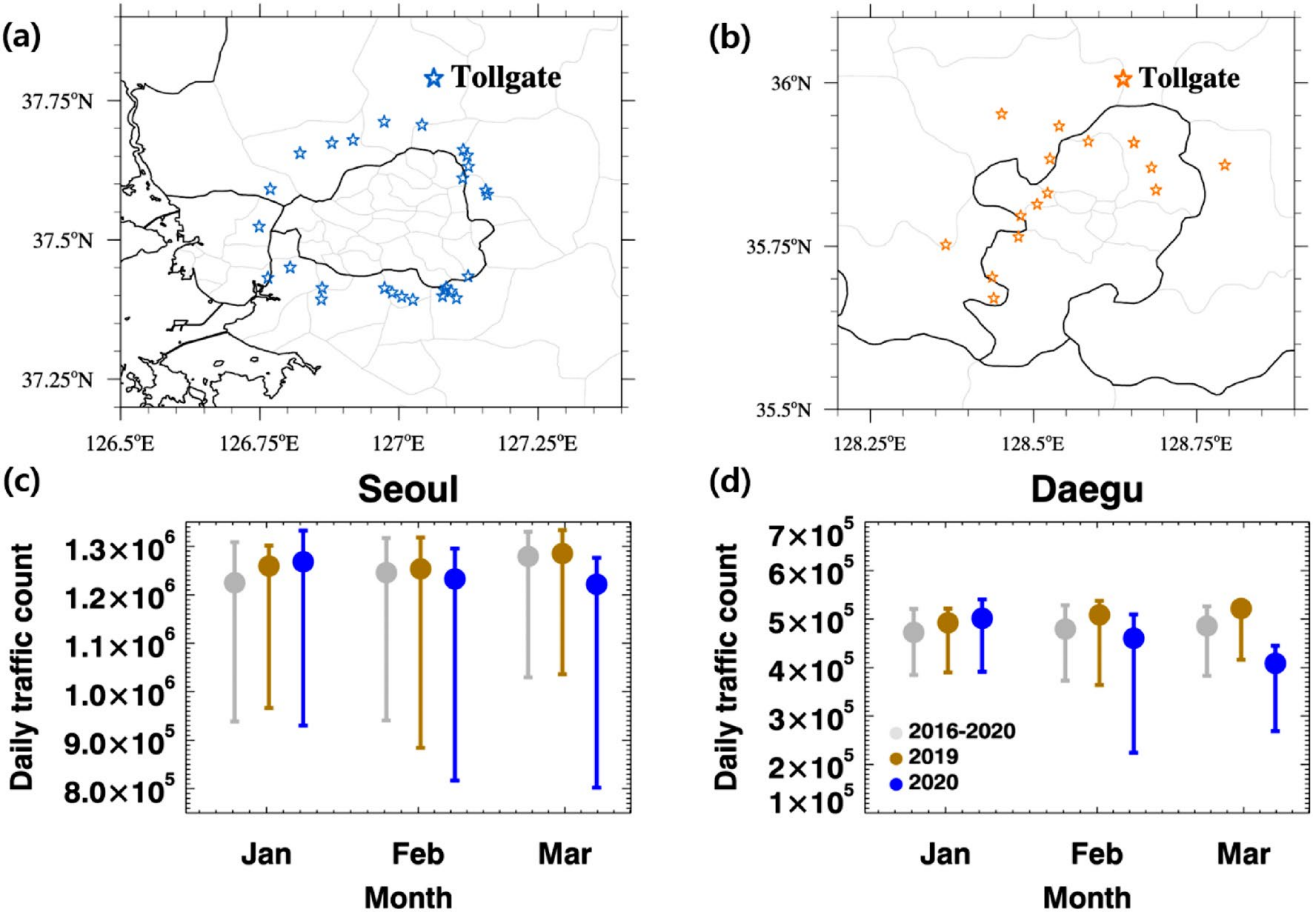
The location of tollgates around (**a**) Seoul and (**b**) Daegu considered in this study. Also, January, February, and March means of the daily traffic count for the entrances and exits of (**c**) Seoul and (**d**) Daegu for 3 periods: 2016–2020, 2019, and 2020. Figure was produced using the NCAR Command Language (NCL) version 6.4.0, which is opened to the public. Download is available at the next website: http://www.ncl.ucar.edu/Download/. For the map information of these figures, we used the geospatial Data provided from the National Geographic Information Institute in South Korea (NGII, ). Data download is available at the next website: http://data.nsdi.go.kr/dataset?q=%ED%96%89%EC%A0%95&sort=score+desc%2C+views_total+de479sc.

We already indicated the difference between the AOD and NO_2_ patterns in South Korea and confirmed that the NO_2_ VCD in Seoul and Daegu became lower compared with the mean pattern (Fig. 2). However, the decreasing extent of NO_2_ VCDs was not quite significant, and even some regions revealed higher NO_2_ values in 2020 (Fig. 1). Surface NO_2_ pattern also revealed this insignificant decrease (Fig. S19). Since traffic emission is the main source of NO_2_^31^ and the NO_2_ variation actually correlates with the vehicle emission well^32^, we also explored the total amount of traffic at the interstate tollgates of Seoul and Daegu (Fig. 5). Interestingly, the traffic in 2020 was more than in previous years in January, but less in February and March, showing that social distancing was working in South Korea. While this reduced traffic connected to the decline in the NO_2_ VCDs in Seoul and Daegu in 2020, the reduction of about a hundred thousand vehicles did not look enough to lead the large decrease of NO_2_.

The large extent of NO_2_ reduction rates in eastern China (Figs. S10 and S11) also well indicate that the traffic decrease in South Korea was relatively small. This speculation is reasonable because the South Korean government did not force city lockdowns or block interstate and international visits despite the serious increase of COVID-19 confirmed cases. Except some restrictions of partial prohibition for entertainment areas and onsite works at schools/companies, the rest of common activities were maintained under spontaneous social distancing. Particularly, commercial distribution industries ordinarily continued, which implies that most of the traffic decrease just resulted from the contraction of private trips. In brief, the small NO_2_ decrease in South Korea indicated that the Korean society normally operated common activities in spite of the spread of COVID-19.

In contrast, the AOD and PM_2.5_ in South Korea had obvious decreases after the COVID-19 outbreak (Figs. 2 and S19). The improvement of the aerosol pollution without a significant NO_2_ change reveals the strong contribution of external factors to the domestic aerosol pollution in South Korea. A study for February and March 2019 indicated the dominant contribution of transboundary transport to the haze in the Korean peninsula^33^. This feature is simply confirmed with the general pattern of back-trajectories obtained from the Hybrid Single-Particle Lagrangian Integrated Trajectory (HYSPLIT) model^34^ calculation (Fig. S20). Figure 1 and S5 also depicted that the progression of the AOD from January to March was quite analogous between South Korea and eastern China. Consequently, it is reasonable to assume that the better air quality in South Korea relates to the better air quality in China. However, again, the enhanced air quality in March 2020 was not only affected by the emission control, but also affected by the meteorology influence. We need to take into consideration that the 90th percentile of the AOD and PM_2.5_ in March 2020 was remarkably lower than in other periods (Figs. 2 and S19), showing that the little occurrence of serious haze is due to the strong ventilation and mixing of local atmospheres. Once more, the consideration of the regional meteorology patterns is essential to avoid exaggerated analyses.

## Summary and conclusion

This study investigated the air quality change in South Korea from January to March 2020 in terms of the influence from the COVID-19 outbreak. The key finding was the improvement of the air pollution in February 2020 when the regional air condition was exceptionally stagnant in recent 5 years, showing the occurrence of large decrease in the regional emissions. However, the enhancement of air quality in South Korea was not quite drastic because social activities were normally retained. Nevertheless, this feature implies that the aggravation of the atmospheric environment can at least be avoided with only a little toleration of small inconveniences such as spontaneous social distancing. In March 2020, the regional air became well mixed and ventilated, resulting in a larger decrease of air pollutants. Based on these analyses, we found that the simple diagnosis without the consideration of regional meteorology can overstate what really happened. Also, a prudent diagnosis will be required for further research of the COVID-19 effect on the regional air quality. The accumulation of these studies shall provide a number of ideas that may enable us to prepare the sustainable development of our society. In that sense, the present COVID-19 era may be a good timing to turn a crisis into a great opportunity.

## Methods

### GOCI AOD

AOD has been widely used for various analyses of aerosol pollution such as the air turbidity and atmospheric composition. In this study, we utilized AOD as the indicator of aerosol loading extent, which is the general idea for the usage of AOD. The geostationary ocean color imager (GOCI) is the instrument onboard the communication, ocean, and meteorological satellite (COMS), which was launched in 2010. The GOCI covers the regions in East Asia (2500 × 2500 km^2^) with a 500 × 500 m^2^ spatial resolution, and it performs hourly measurements from 00:30 to 07:30 UTC (09:30–16:30 Korean Standard Time)^35^. Various aerosol properties were retrieved by the Yonsei aerosol retrieval (YAER) algorithm using six visible and two near-infrared channels of the GOCI^36–38^. Among them, we utilized the AOD at the 550-nm channel, which was well validated it other ground-based and satellite observations^39^. To obtain better data quality, we reproduced daily AOD values with the 6 × 6 km^2^ spatial resolution (Fig. S21) and then estimated the monthly mean AOD. To avoid the contamination of the snow-covered surfaces, the monthly mean was calculated when the AOD data was available for longer than at least 3 days per month, following the algorithm theoretical basis documents (ATBD) of the moderate resolution imaging spectroradiometer (MODIS) mission^40^. For the regional analysis, we averaged all AOD values in the administrative districts obtained from the geographic information system.

### OMI NO_2_ VCD

The ozone monitoring instrument (OMI) is the ultraviolet–visible spectrometer (270–500 nm with 0.4–0.6 nm spectral resolution) onboard the Aura satellite, which was launched in 2004^41,42^. OMI has a 13 × 24 km^2^ spatial resolution in a nadir viewing observation with 60 cross-track positions (Fig. S21), and the local overpassing time is at 13:30–14:00. In this study, we used the OMI NO_2_ Standard Product (OMNO2) version 4.0, which was provided by the NASA Goddard space flight center^43^. The OMNO2 algorithm first retrieved the slant column of the NO_2_ by using the differential optical absorption spectroscopy (DOAS) method. Then, it calculated the total NO_2_ VCD by applying the air mass factor (AMF). Finally, the tropospheric NO_2_ VCD was obtained by subtracting the stratospheric portions from the total NO_2_ VCDs^44^. Compared with the previous version^45^, the OMNO2 version 4.0 algorithm first applied the data of the geometry-dependent surface Lambertian Equivalent Reflectivity^46^ for the AMF calculations. The OMI experienced the row anomaly problem since 2007 (https://projects.knmi.nl/omi/research/product/rowanomaly-background.php), which affects the quality of the radiance measurements for some cross-track positions. In this study, we excluded all affected pixels by the row anomaly for the analysis. To avoid strange values, we also used the OMNO2 products that have a proper quality flag (the least significant bit of VcdQualityFlags parameter equal to 0), a cloud radiance fraction < 0.5, and a solar zenith angle < 70°. For the calculation of the gridded monthly mean data of tropospheric NO_2_ VCDs, we oversampled the OMI observations by applying the tessellation approach^47,48^, which provides area- and uncertainty-weighted average NO_2_ columns. For the oversampling of the OMI data, a grid resolution of 0.05° × 0.05° was used (Fig. S21).

### TROPOMI NO_2_ VCD

The tropospheric Monitoring Instrument (TROPOMI) is a spectrometer onboard the Sentinel-5 Precursor satellite, which was launched in 2017, for measuring the solar and backscattered earthshine radiation at ultraviolet–visible (270–500 nm), near-infrared (710–770 nm), and shortwave infrared (2314–2382 nm) wavelengths^49^. Its local overpassing time was 13:30–14:00, and the spatial resolution was initially 3.5 × 7.0 km^2^, but it was improved to 3.5 × 5.5 km^2^ in August 2019 (Fig. S21). The standard algorithm of the TROPOMI offline NO_2_ product, which was processed by the Royal Netherlands Meteorological Institute (KNMI), first retrieved the slant column using the DOAS method and then calculated the NO_2_ VCD by applying the AMF. Finally, it obtained the tropospheric NO_2_ VCDs^50–52^. For qualified analyses, we used the tropospheric NO_2_ VCDs, which have a quality assurance value (qa_value > 0.75). The tessellation approach was also applied for the estimation of the gridded monthly averages of the tropospheric NO_2_ VCDs from the TROPOMI with a grid resolution of 0.02° × 0.02° (Fig. S21).

### AIRKOREA surface measurement

The surface observed PM_2.5_, NO_2_, and ozone in South Korea were obtained from the AIRKOREA data archive (https://www.airkorea.or.kr/web) provided by the National Ambient air quality Monitoring Information System in South Korea. The PM_2.5_ (unit: μg/m^3^) was measured using the β-ray absorption method, which utilizes the relationship between the extent of the β-ray absorption and the amount of the filtered PM_2.5_. The NO_2_ (unit: ppb) was measured using the chemiluminescent method, which detects the extent of the chemiluminescence from the reaction between the NO and ozone. The ozone (unit: ppb) was measured by the ultraviolet photometric method, which utilizes the relationship between the ultraviolet absorption extent and the ozone amount. There is a total of 25 monitoring sites in Seoul and 14 in Daegu. The monthly mean values were estimated using the hourly data from all sites in each city.

### ERA5 meteorology data

For the analysis of the regional meteorological pattern, we used the wind field and air temperature values obtained from the newest reanalysis dataset of the European Centre for Medium-Range Weather Forecasts, ERA5, which provides hourly meteorology variables since 1950 through the Copernicus climate change service^53^. ERA5 has a 31 km horizontal resolution and vertically 137 atmospheric layers from the surface to 80 km altitude. For the wind pattern analysis, we calculated the wind speed index (WSI), which describes the relative strength of the wind speed in a target period compared with the climatological average. We compared the monthly mean WSI for multiple years using Eq. (1).

1 $${WSI}_{i}^{j}=\frac{{WS}_{i}^{j}-{WS}_{mean}^{j}}{{WS}_{std}^{j}},$$

where *i* is the year, *j* is the number of pixels, $W{S}_{i}^{j}$ is the monthly mean wind speed of the *i* year and *j* pixel, and $W{S}_{mean}^{j}$ and $W{S}_{std}^{j}$ denote the mean and standard deviation of the monthly mean wind speed of the *j* pixel for the recent 20 years (from 2000 to 2020), respectively. Additionally, we calculated the air temperature gradient index (ATGI), which describes the vertical stability using the air temperature between two altitudes. Similar to the WSI calculation, the ATGI is calculated as in Eq. (2).

2 $${ATGI}_{i}^{j}=\frac{{ATG}_{i}^{j}-{ATG}_{mean}^{j}}{{ATG}_{std}^{j}},$$

where *ATG* is the air temperature gradient, which is the air temperature (*AT*) difference between the 925 and 1000 hPa altitudes ($ATG={AT}_{925hPa}-{AT}_{1000hPa}$) for the *i* year and *j* pixel. Also, ${ATG}_{mean}^{j}$ and ${ATG}_{std}^{j}$ denote the mean and standard deviation of the ATG of the *j* pixel for the recent 20 years (from 2000 to 2020). Thus, a positive (negative) ATGI signifies a vertically stable (unstable) air condition.

### HYSPLIT model

The Hybrid Single-Particle Lagrangian Integrated Trajectory (HYSPLIT) was developed by the Air Resources Laboratory (ARL) in the National Oceanic and Atmospheric Administration (NOAA)^34^. This model have been widely used for the calculation of forward- and back-trajectories to examine the transport pattern of air masses. In this study, we used the HYSPLIT model to calculate the 48-h back-trajectories per every hour arrived at Seoul and Daegu, South Korea from 1 January to 31 March 2020. Gridded meteorology data (1 × 1°) from the Global Data Assimilation System (GDAS) were used for this calculation.

### Traffic data

The Korea Expressway Corporation is a public enterprise for the management of all the expressways in South Korea. They count the traffic at each tollgate (the number of vehicle access) and provide daily total traffic data on their web-archive (http://data.ex.co.kr/). To estimate the daily traffic data in Seoul and Daegu, we selected all the tollgates in the 20 km range from the center of each city (Seoul: 37.57° N, 126.76° E, Daegu: 35.79° N, 128.65° E). As a result, 27 and 16 tollgates were selected at Seoul and Daegu, respectively.

## Supplementary Information

Supplementary Information

## Data Availability

Traffic information is open to the public. Data download is available at the next website: http://data.ex.co.kr/. HYSPLIT model is open to the public. Model (HYSPLIT version 4 offline model) download is available at the next website: https://www.ready.noaa.gov/HYSPLIT_hytrial.php. Meteorology data (GDAS 1 degree data) download for the model simulation is available at the next website: https://www.ready.noaa.gov/archives.php. OMI data are available from NASA GES DISC (Goddard Earth Sciences Data and Information Services Center) https://aura.gesdisc.eosdis.nasa.gov/data/Aura_OMI_Level2/OMNO2.003/. TROPOMI data are avaiable from NASA GES DISC https://tropomi.gesdisc.eosdis.nasa.gov/data/S5P_TROPOMI_Level2/. GOCI aerosol data used in this work are available upon request to the corresponding author.
