# Supplementary material for: The implication of the air quality pattern in South Korea after the COVID-19 outbreak
Source: Sci Rep. 2020 Dec 31;10:22462. doi: 10.1038/s41598-020-80429-4 (PMC7775425; doi:10.1038/s41598-020-80429-4)
Supplement: Supplementary file 1 — Supplementary Information [file 41598_2020_80429_MOESM1_ESM.docx]

**[Supplementary Information]**

**The Implication of the Air Quality Pattern in South Korea**

**after the COVID-19 Outbreak**

Ja-Ho Koo^1^, Jhoon Kim^1*^, Yun Gon Lee^2*^, Sang Seo Park^3^, Seoyoung Lee^1^,

Heesung Chong^1^, Yeseul Cho^1^, Jaemin Kim^2^, Kyungbae Choi^2^, and Taegyung Lee^1^

^1^Department of Atmospheric Sciences, Yonsei University, Seoul, Republic of Korea

^2^Department of Atmospheric Sciences, Chungnam National University, Daejeon, Republic of Korea

^3^School of Urban and Environmental Engineering, Ulsan National Institute of Science and Technology, Ulsan, Republic of Korea

*Corresponding author: Jhoon Kim ([jkim2@yonsei.ac.kr](mailto:jkim2@yonsei.ac.kr)), Yun Gon Lee (yglee2@cnu.ac.kr)


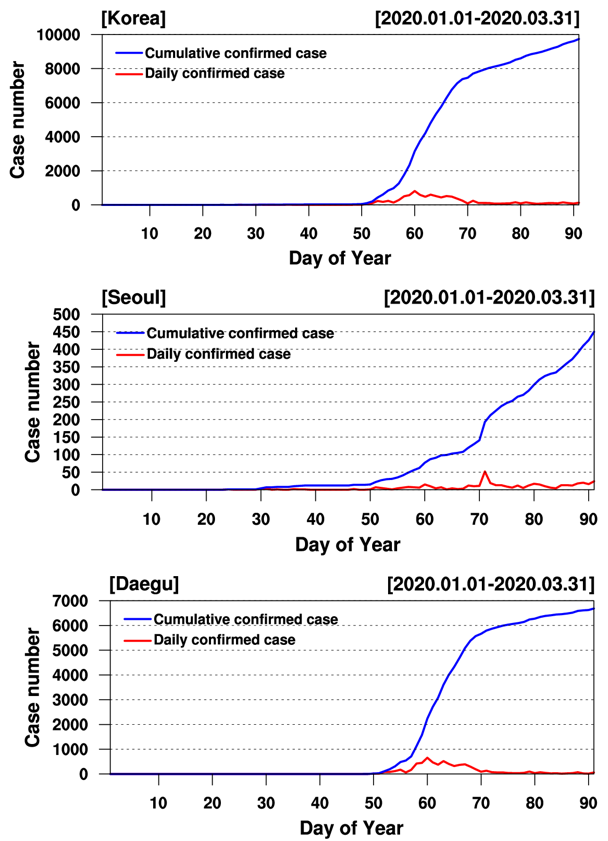


**Figure S1.** Situation of the COVID-19 cases from 1 January to 31 March 2020 in the whole of South Korea (top), Seoul (middle), and Daegu (bottom). The daily (red) and cumulative (blue) confirmed cases of COVID-19 are suggested.


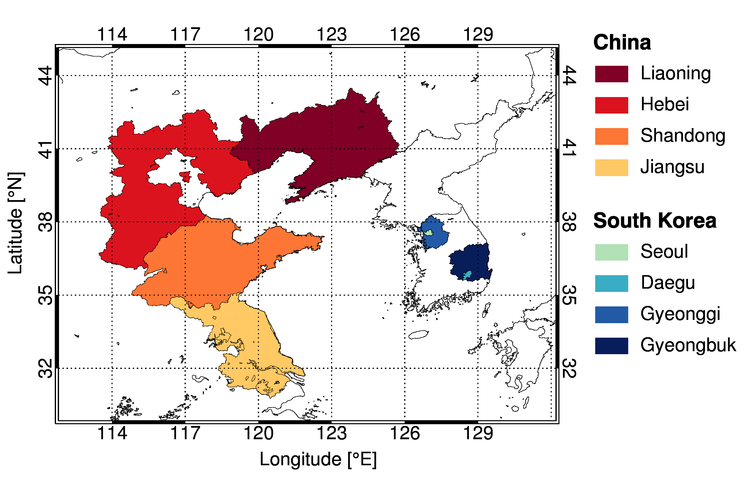


**Figure S2.** A map showing the target regions in this study. In South Korea, we investigated the air quality pattern in the Gyeonggi province surrounding the city of Seoul and in the Gyeongbuk province surrounding the city of Daegu. In China, we focused on 4 provinces closely located to the Korean peninsula: Liaoning, Hebei, Shandong, and Jiangsu. The district geometries in South Korea are available from <http://www.gisdeveloper.co.kr/?p=2332>. The district geometries in China are available from http://www.gadm.org/.


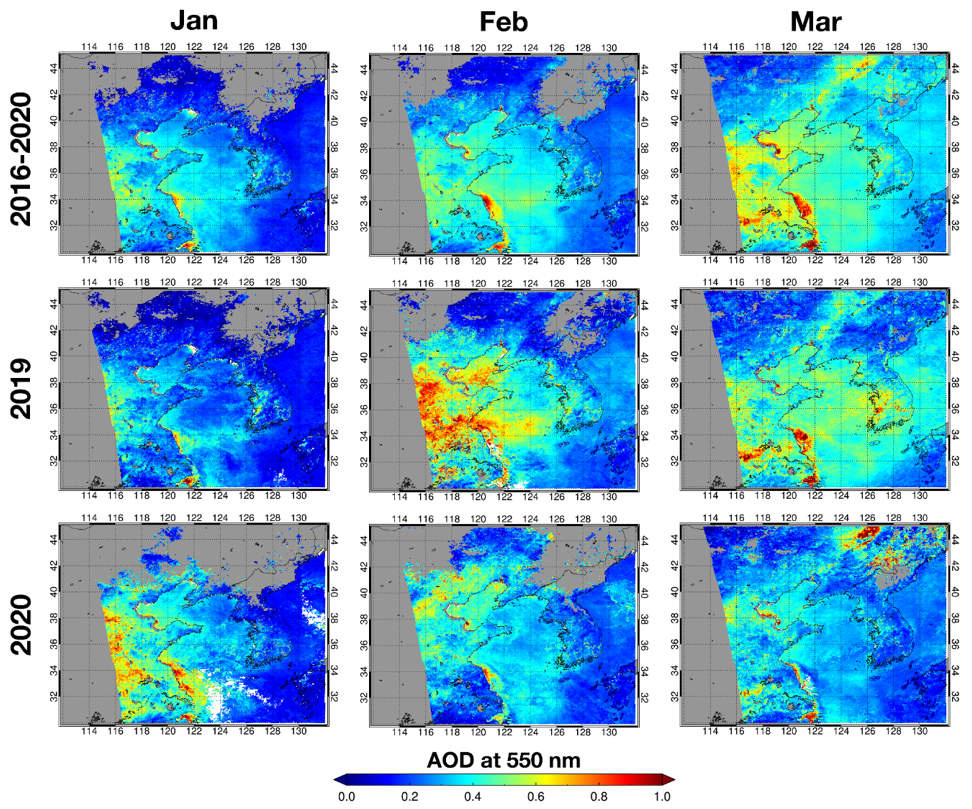


**Figure S3.** Average GOCI AODs at 550 nm in East Asia in January (left), February (middle), and March (right) for the periods of 2016–2020, 2019, and 2020. Figures generated with Interactive Data Language (IDL) version 8.8.0 (https://www.l3harrisgeospatial.com/Software-Technology/IDL).


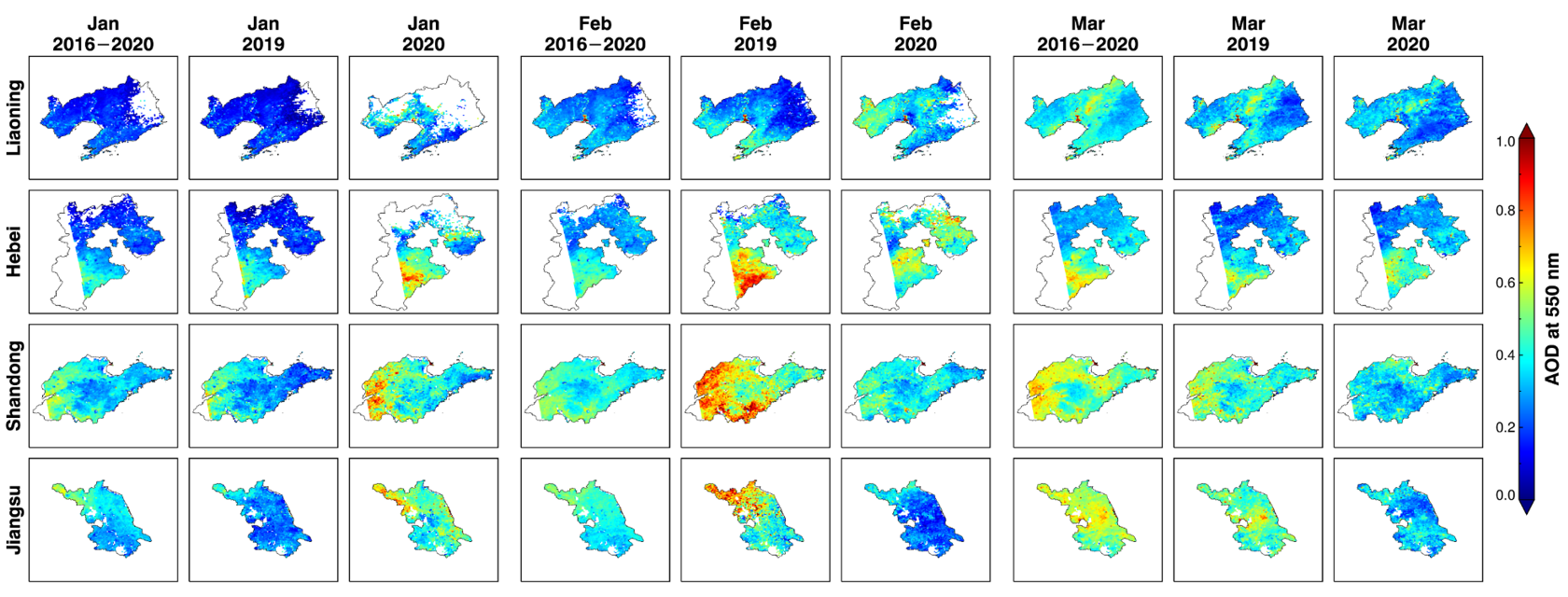


**Figure S4.** Monthly average GOCI AODs at 550 nm in 4 provinces located in eastern China (Liaoning, Hebei, Shandong, and Jiangsu) in January (left panel), February (middle panel), and March (right panel) for the periods of 2016–2020, 2019, and 2020. The district geometries in China are available from http://www.gadm.org/.


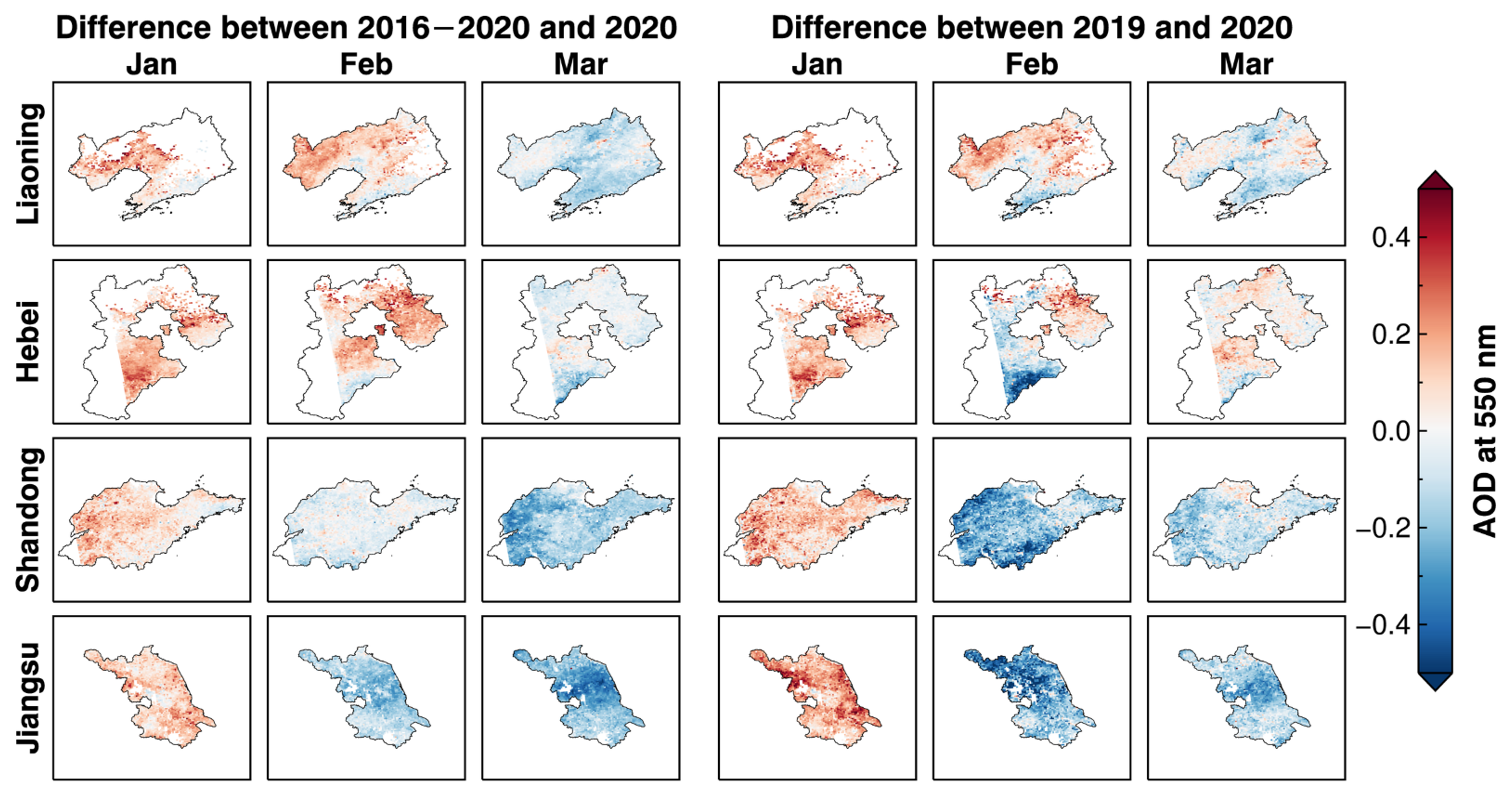


**Figure S5.** The change in the average GOCI AODs at 550 nm in January, February, and March in 2020 over the Liaoning, Hebei, Shandong, and Jiangsu provinces, China, compared with those during recent 5 years (2016–2020) (left) and in 2019 (right). Blue color scale indicates the lower AOD in 2020, and red color scale indicates the higher AOD in 2020. The district geometries in China are available from http://www.gadm.org/.


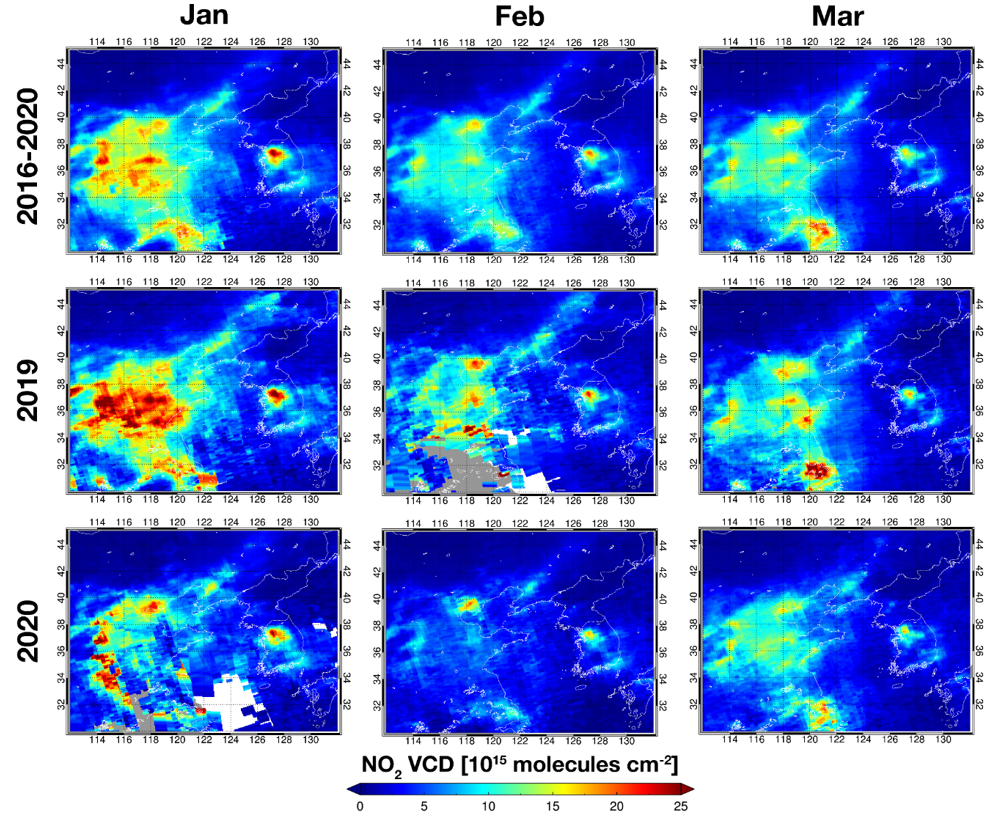


**Figure S6.** The average OMI NO_2_ VCDs in East Asia in January (left), February (middle), and March (right) for the periods of 2016–2020 (upper), 2019 (middle), and 2020 (lower panel). Figures generated with Interactive Data Language (IDL) version 8.8.0 (https://www.l3harrisgeospatial.com/Software-Technology/IDL).


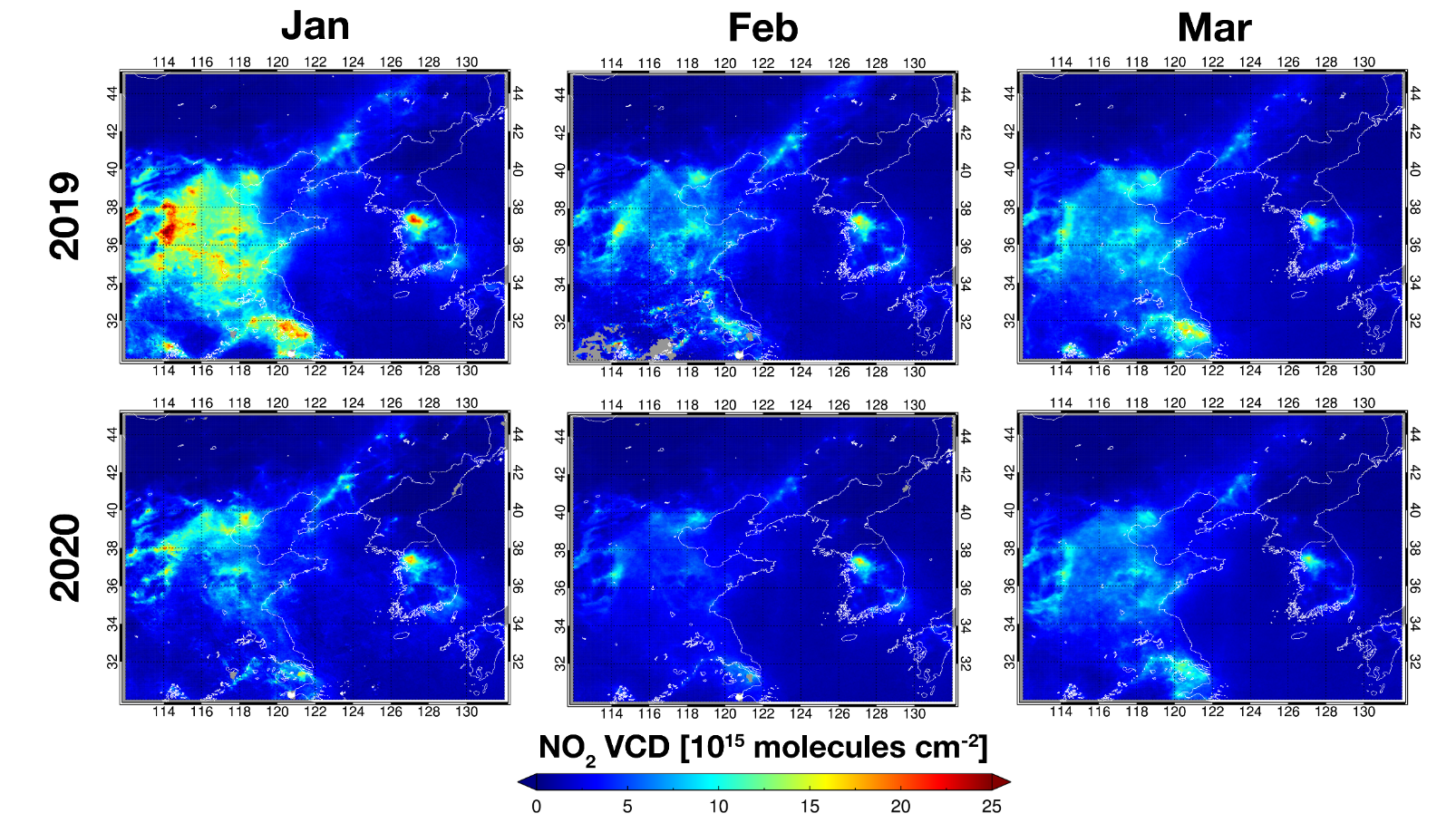


**Figure S7.** Average TROPOMI NO_2_ VCDs in East Asia in January (left), February (middle), and March (right) for the years of 2019 and 2020. Figures generated with Interactive Data Language (IDL) version 8.8.0 (https://www.l3harrisgeospatial.com/Software-Technology/IDL).


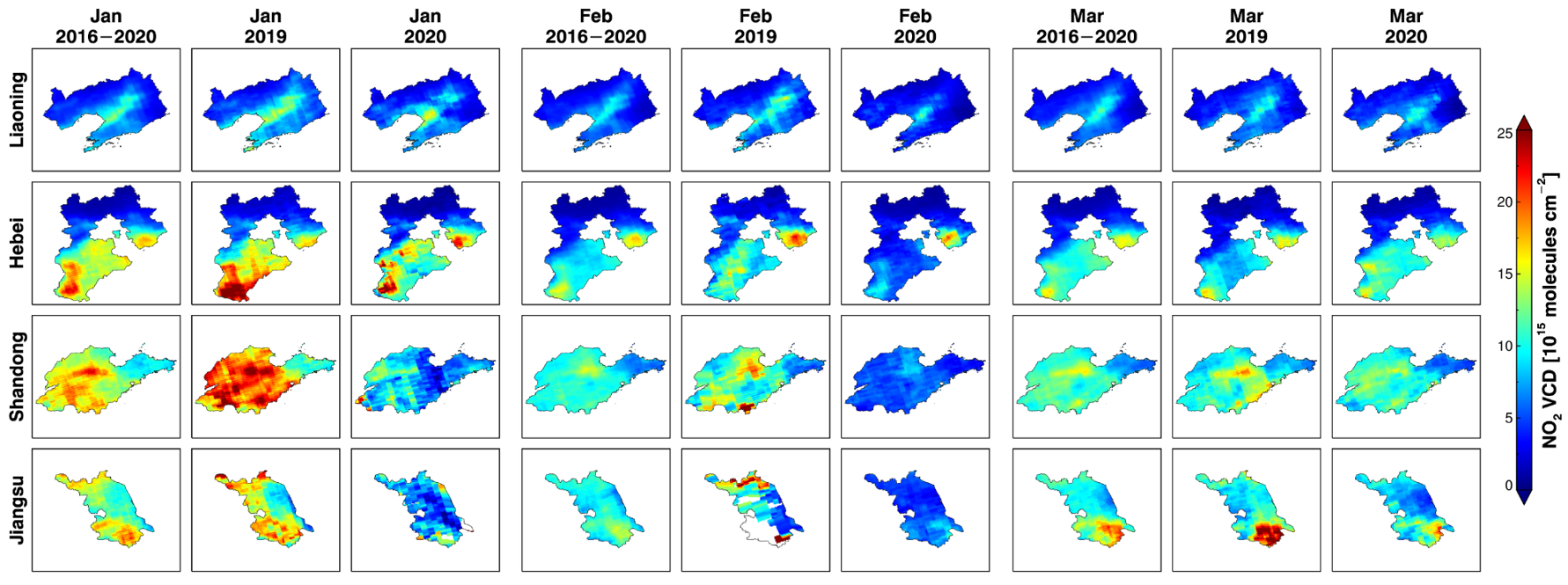


**Figure S8.** Average OMI NO_2_ VCDs in 4 provinces located in eastern China (Liaoning, Hebei, Shandong, and Jiangsu) in January (left panel), February (middle panel), and March (right panel) for the periods of 2016–2020, 2019, and 2020. The district geometries in China are available from http://www.gadm.org/.


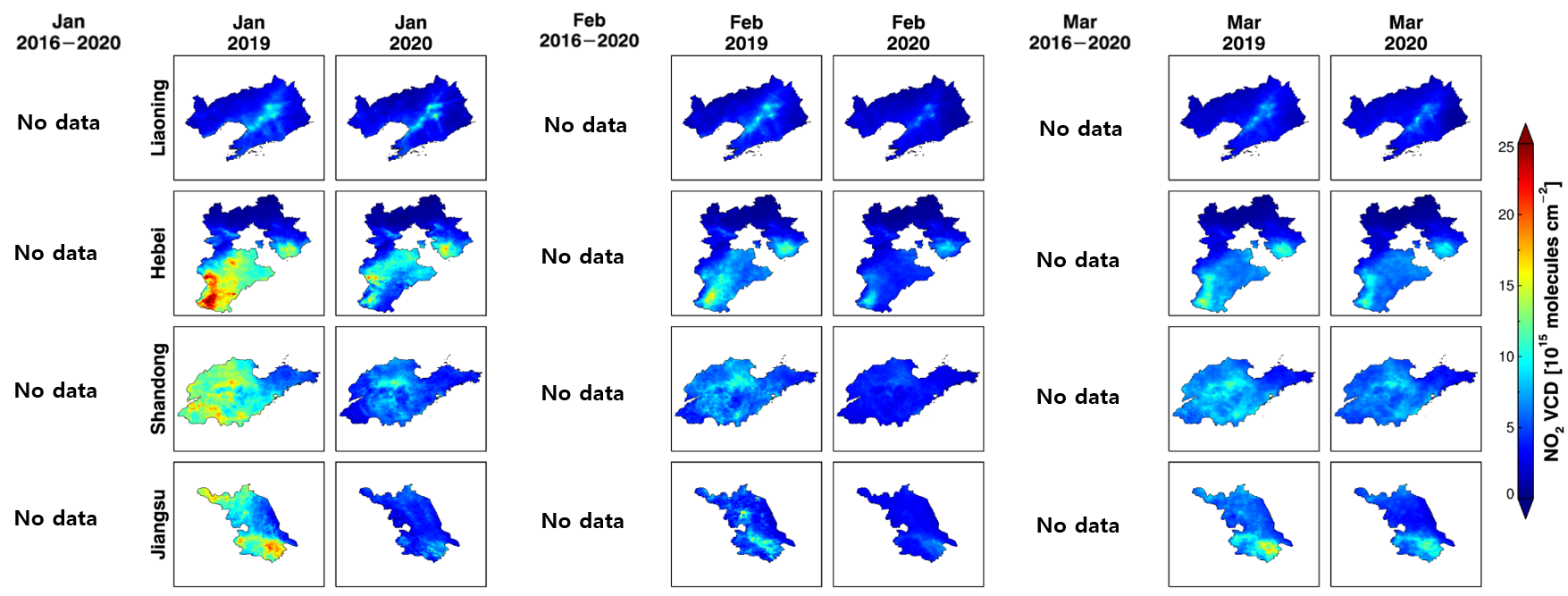


**Figure S9.** Average TROPOMI NO_2_ VCDs in 4 provinces located in eastern China (Liaoning, Hebei, Shandong, and Jiangsu) in January (left panel), February (middle panel), and March (right panel) for the periods of 2019 and 2020. The district geometries in China are available from http://www.gadm.org/.


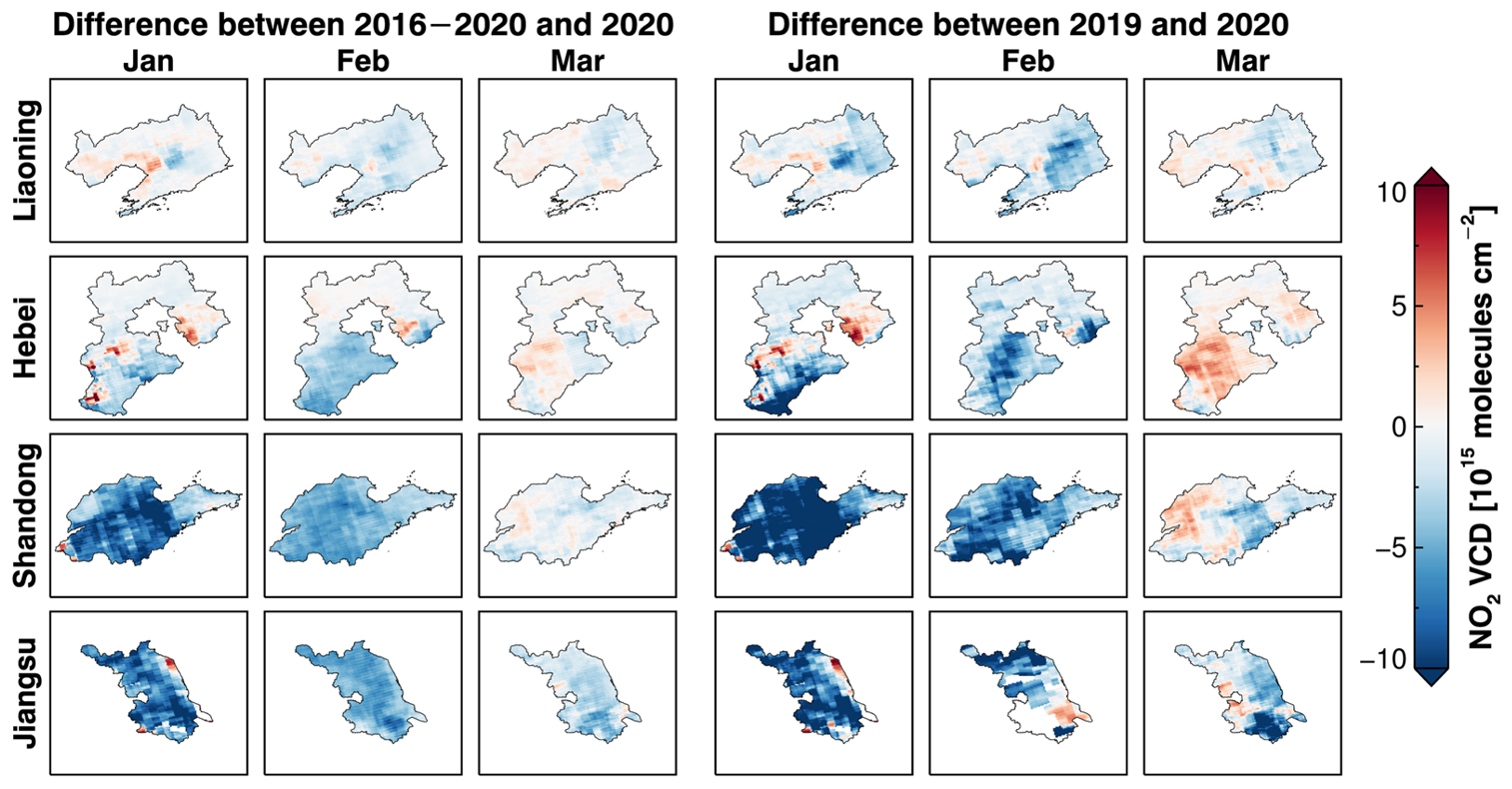


**Figure S10.** The change in the January, February, and March means of the OMI NO_2_ VCD in 2020 over the Liaoning, Hebei, Shandong, and Jiangsu provinces, China, compared with the January, February, and March means of the OMI NO_2_ VCD during recent 5 years (2016–2020) (left) and in 2019 (right). Namely, the blue color scale indicates the smaller NO_2_ VCD in 2020, and the red color scale indicates the larger NO_2_ VCD in 2020. The district geometries in China are available from http://www.gadm.org/.


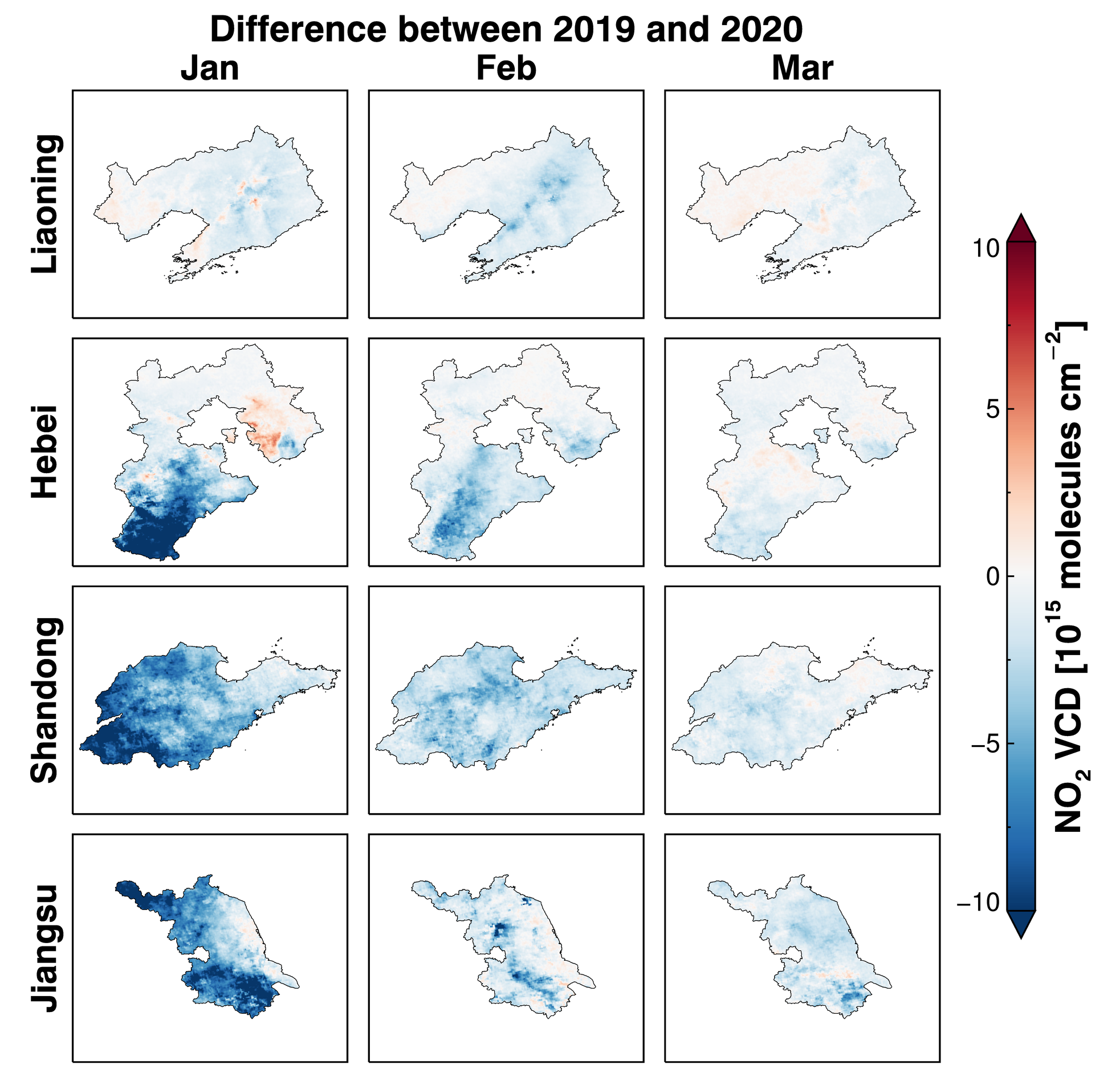


**Figure S11.** The change in the January, February, and March means of the TROPOMI NO_2_ VCD in 2020 over the Liaoning, Hebei, Shandong, and Jiangsu provinces, China, compared with the January, February, and March means of the TROPOMI NO_2_ VCD in 2019. Namely, the blue color scale indicates the smaller NO_2_ VCD in 2020, and the red color scale indicates the larger NO_2_ VCD in 2020. The district geometries in China are available from http://www.gadm.org/.


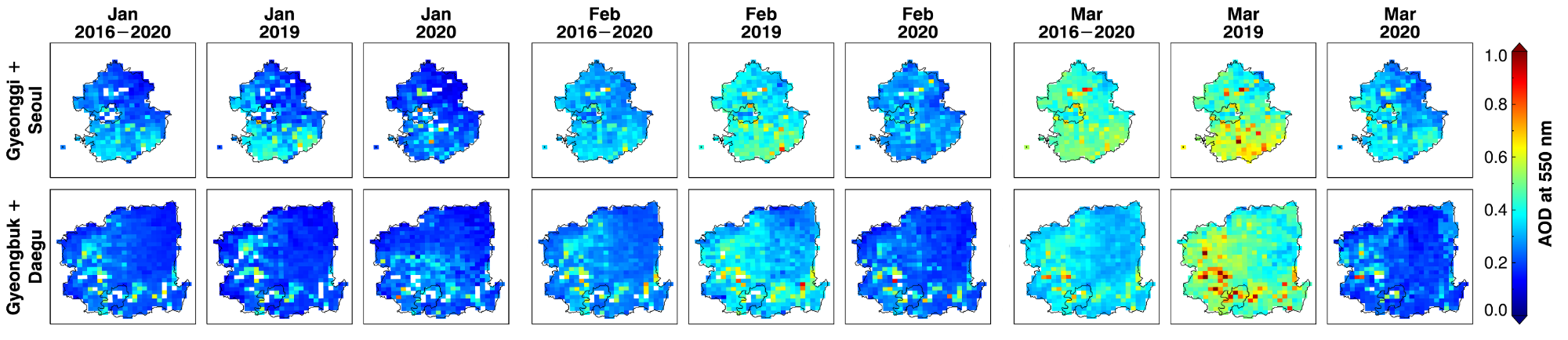


**Figure S12.** Average GOCI AODs at 550 nm in the Gyeonggi province surrounding Seoul (top) and in the Gyeongbuk province surrounding Daegu (bottom) in January (left panel), February (middle panel), and March (right panel) for the periods of 2016–2020, 2019, and 2020. The district geometries in South Korea are available from http://www.gisdeveloper.co.kr/?p=2332.


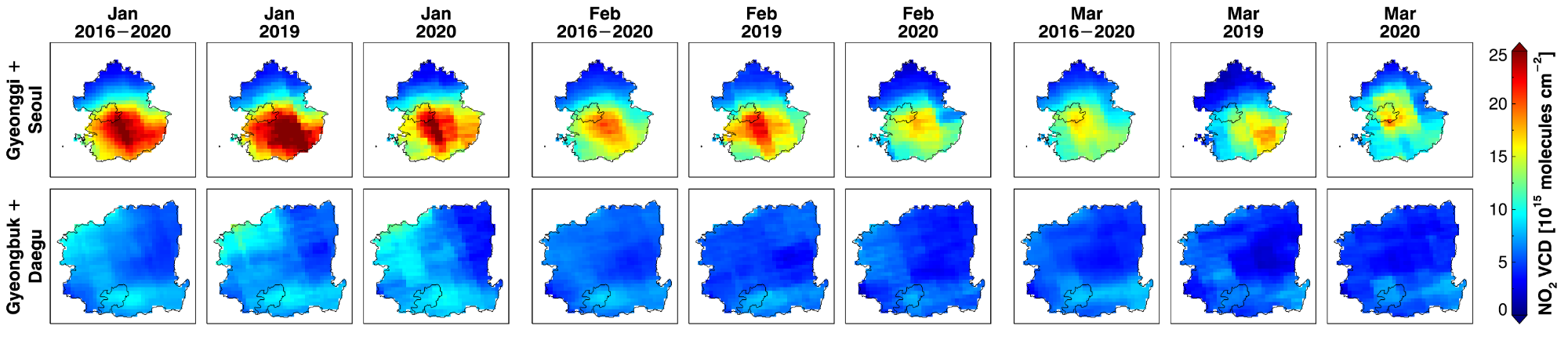


**Figure S13.** Average OMI NO_2_ VCD in the Gyeonggi province surrounding Seoul (top) and in the Gyeongbuk province surrounding Daegu (bottom) in January (left panel), February (middle panel), and March (right panel) for the periods of 2016–2020, 2019, and 2020. The district geometries in South Korea are available from http://www.gisdeveloper.co.kr/?p=2332.


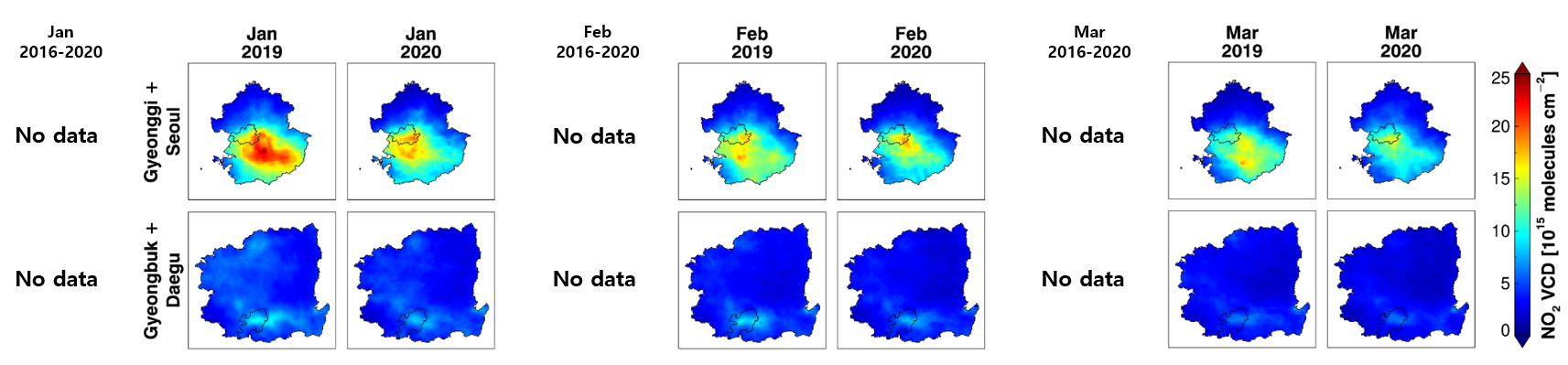


**Figure S14.** Average TROPOMI NO_2_ VCDs in the Gyeonggi province surrounding Seoul (top) and in the Gyeongbuk province surrounding Daegu (bottom) in January (left panel), February (middle panel), and March (right panel) for the periods of 2016–2020, 2019, and 2020. The district geometries in South Korea are available from http://www.gisdeveloper.co.kr/?p=2332.


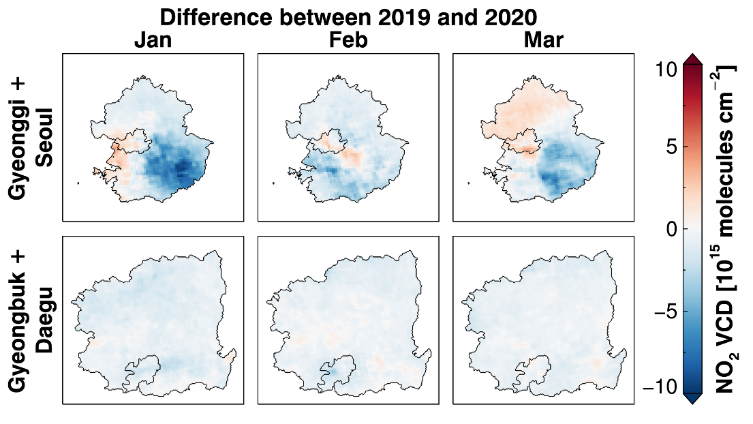


**Figure S15.** The change in the January, February, and March means of the TROPOMI NO_2_ VCD (bottom) in 2020 over the Gyeonggi (surrounding Seoul) and Gyeongbuk (surrounding Daegu) provinces, South Korea, compared with the January, February, and March means of the TROPOMI NO_2_ VCD in 2019. Namely, the blue color scale indicates the smaller NO_2_ VCD in 2020, and the red color scale indicates the larger NO_2_ VCD in 2020. The district geometries in South Korea are available from http://www.gisdeveloper.co.kr/?p=2332.


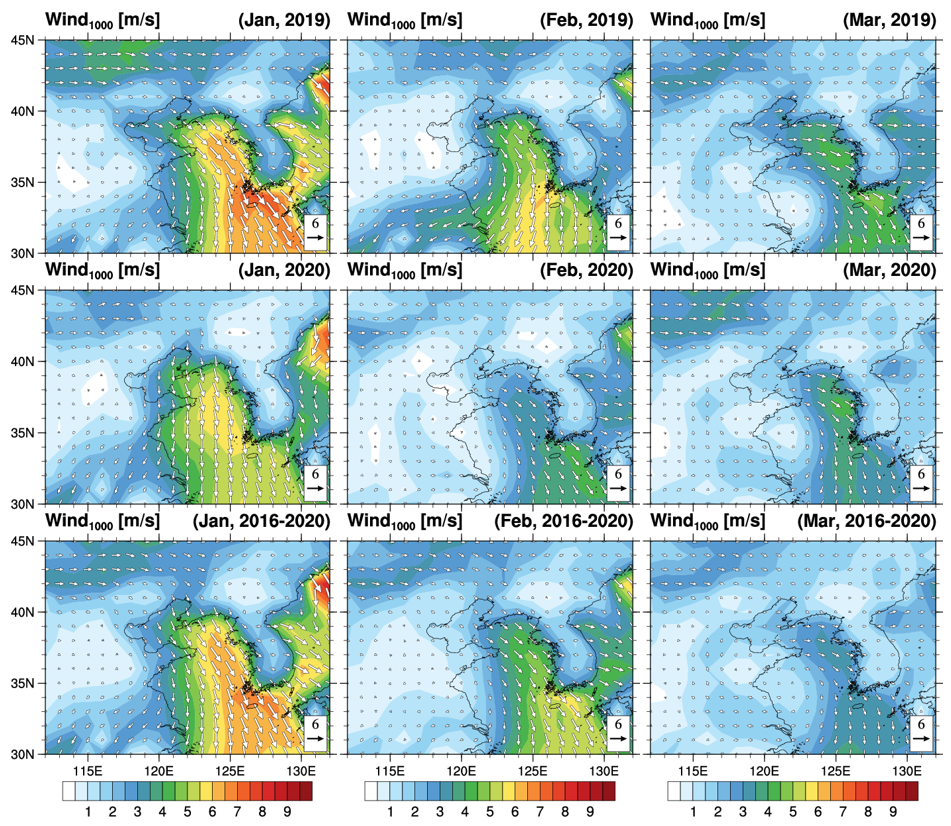


**Figure S16.** January (left), February (middle), and March (right) means of the wind speed at 1000 hPa in East Asia for 3 periods: 2019 (top), 2020 (middle), and 2016–2020 (bottom). The wind direction is illustrated by arrows, and the wind speed is described by both the size of the arrows and the color scale. Figure was produced using the NCAR Command Language (NCL) version 6.4.0, which is opened to the public. Download is available at the next website: http://www.ncl.ucar.edu/Download/. For the map information of these figures, we used the geospatial Data provided from the National Geographic Information Institute in South Korea (NGII, <https://www.ngii.go.kr/eng/main.do>) and Regionally Accessible Nested Global Shorelines (RANGS) coastline database ([https://www.ncl.ucar.edu/Document/Graphics/rangs.shtml)](https://www.ncl.ucar.edu/Document/Graphics/rangs.shtml%29%EB%9D%BC%EB%8A%94). Data can be downloaded from the next website: http://data.nsdi.go.kr/dataset?q=%ED%96%89%EC%A0%95&sort=score+desc%2C+views_total+desc


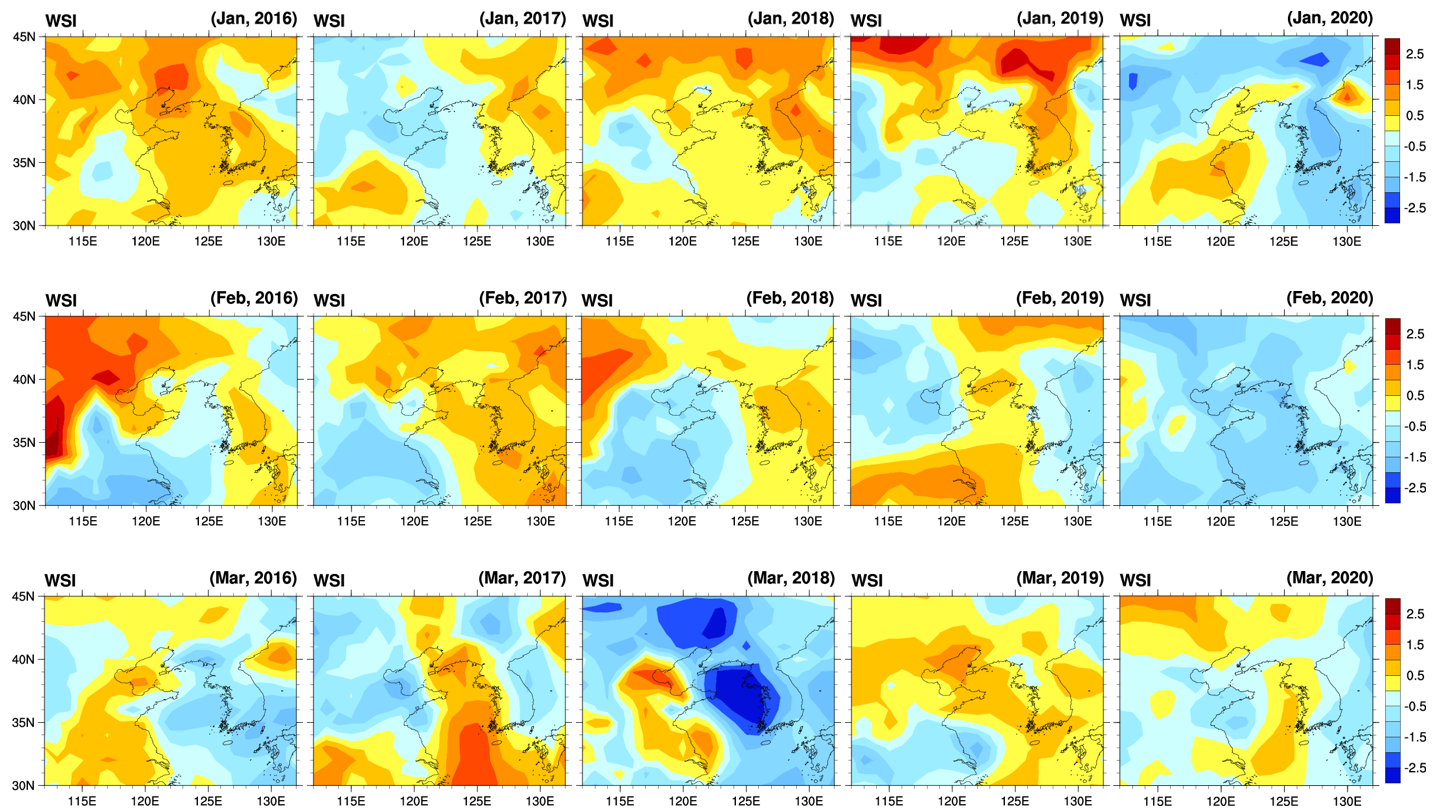


**Figure S17.** January (top), February (middle), and March (bottom) means pattern of the wind speed index (WSI) in East Asia from 2016 to 2020. The red color scales reveal the faster wind speed, and the blue color scales reveal the slower wind speed compared with the climatological wind speed. Figure was produced using the NCAR Command Language (NCL) version 6.4.0, which is opened to the public. Download is available at the next website: http://www.ncl.ucar.edu/Download/. For the map information of these figures, we used the geospatial Data provided from the National Geographic Information Institute in South Korea (NGII, <https://www.ngii.go.kr/eng/main.do>) and Regionally Accessible Nested Global Shorelines (RANGS) coastline database ([https://www.ncl.ucar.edu/Document/Graphics/rangs.shtml)](https://www.ncl.ucar.edu/Document/Graphics/rangs.shtml%29%EB%9D%BC%EB%8A%94). Data download is available at the next website: http://data.nsdi.go.kr/dataset?q=%ED%96%89%EC%A0%95&sort=score+desc%2C+views_total+desc


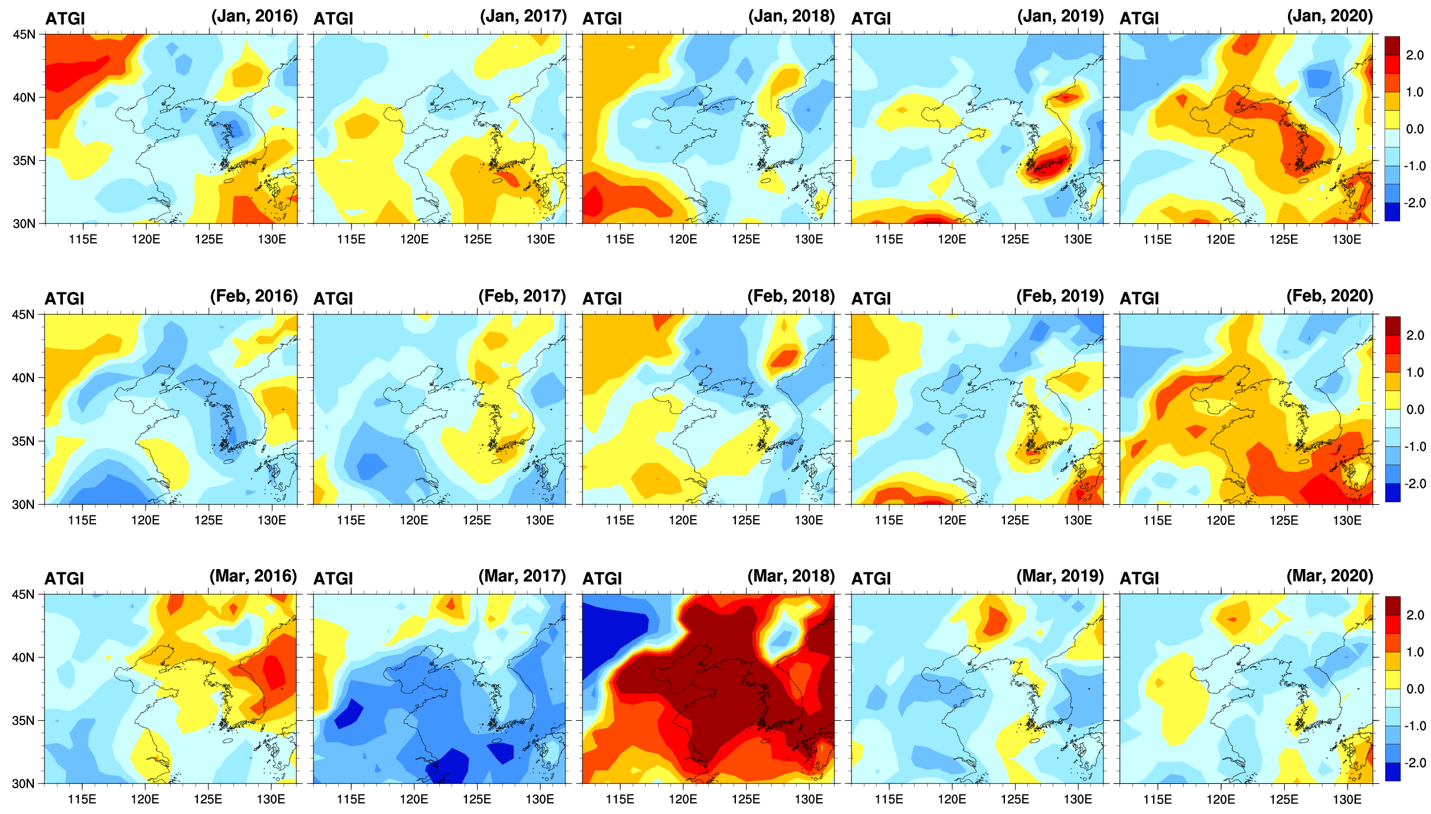


**Figure S18.** January (top), February (middle), and March (bottom) means pattern of the air temperature gradient index (ATGI) in East Asia from 2016 to 2020. The red color scales reveal that the air temperature at 925 hPa is larger than that at 1000 hPa (i.e., vertically stable condition), and the blue color scales reveal that the air temperature at 925 hPa is smaller than that at 1000 hPa (i.e., vertically unstable condition). Figure was produced using the NCAR Command Language (NCL) version 6.4.0, which is opened to the public. Download is available at the next website: http://www.ncl.ucar.edu/Download/. For the map information of these figures, we used the geospatial Data provided from the National Geographic Information Institute in South Korea (NGII, <https://www.ngii.go.kr/eng/main.do>) and Regionally Accessible Nested Global Shorelines (RANGS) coastline database ([https://www.ncl.ucar.edu/Document/Graphics/rangs.shtml)](https://www.ncl.ucar.edu/Document/Graphics/rangs.shtml%29%EB%9D%BC%EB%8A%94) . Data download is available at the next website: http://data.nsdi.go.kr/dataset?q=%ED%96%89%EC%A0%95&sort=score+desc%2C+views_total+desc


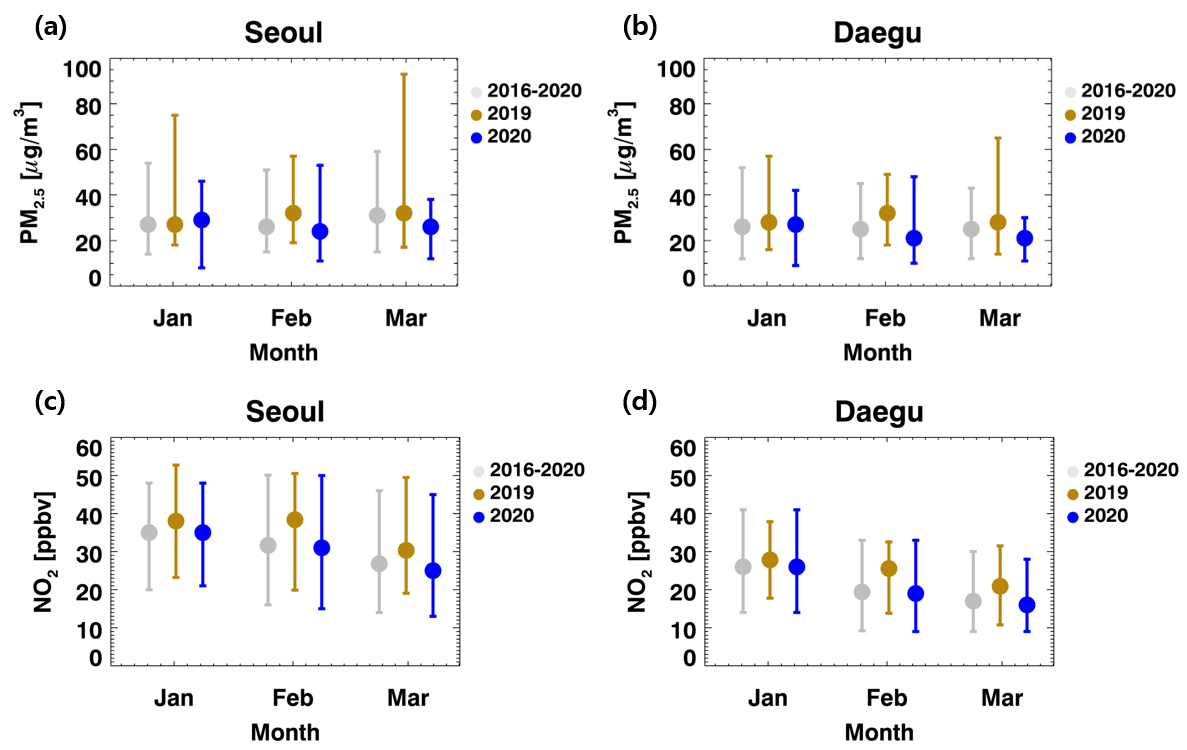


**Figure S19.** Percentiles of the observed surface PM_2.5_ (top) and NO_2_ mixing ratio (bottom) in Seoul (left) and Daegu (right), South Korea for January, February, and March. The 10th, 50th, and 90th percentiles of January, February, and March are compared for 3 periods: from 2016 to 2020 (gray), 2019 (yellow), and 2020 (blue).


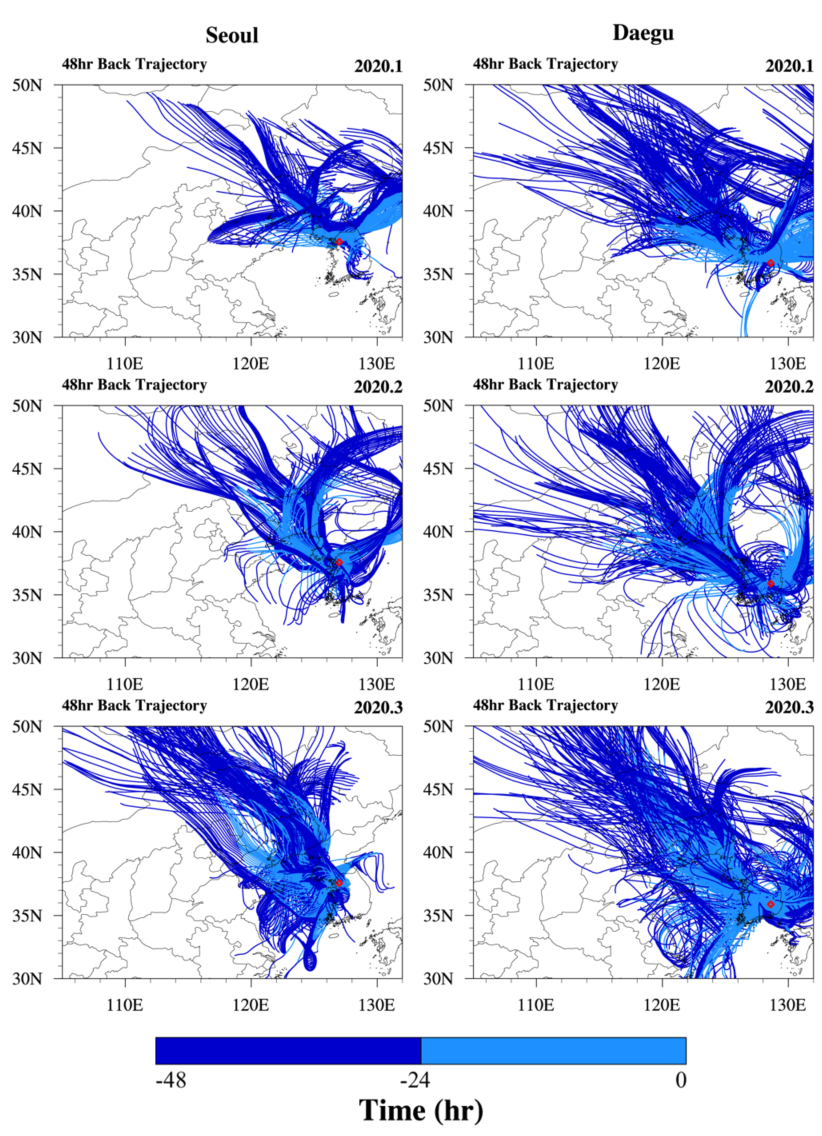


**Figure S20.** 48-hour back-trajectories arrived to Seoul (left) and Daegu (right) during January (top), February (middle), and March (bottom) 2020, calculated by the HYSPLIT model. Light (dark) blue color indicates the air-mass pathway in from 0 to 24 hours (from 24 to 48 hours) before the arrival to Seoul and Daegu. Figure was produced using the NCAR Command Language (NCL) version 6.4.0, which is opened to the public. Download is available at the next website: http://www.ncl.ucar.edu/Download/. For the map information of these figures, we used the geospatial Data provided from the National Geographic Information Institute in South Korea (NGII, https://www.ngii.go.kr/eng/main.do). Data download is available at the next website: http://data.nsdi.go.kr/dataset?q=%ED%96%89%EC%A0%95&sort=score+desc%2C+views_total+desc


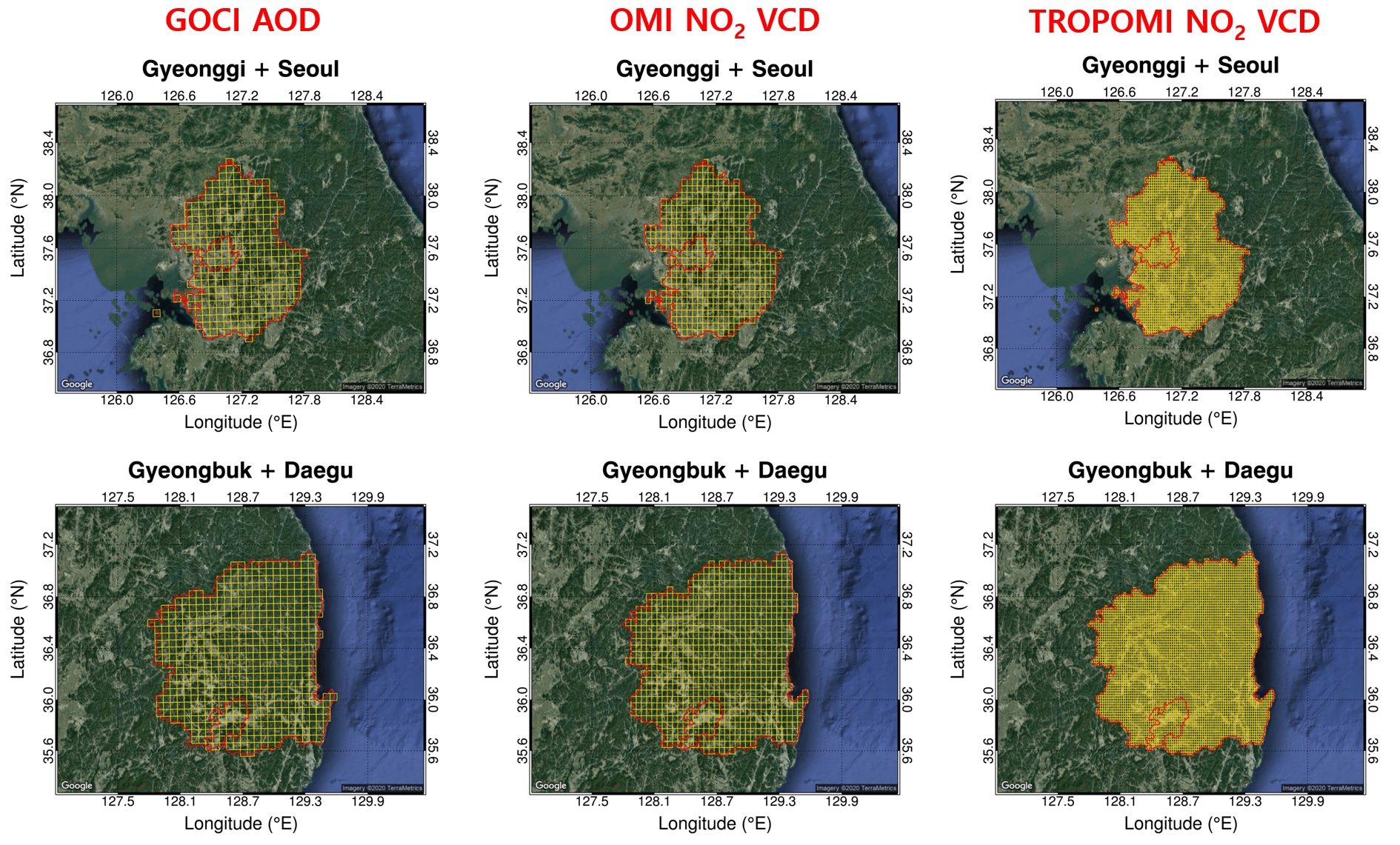


**Figure S21.** Illustration of the pixel distribution of the GOCI AOD (left), OMI NO_2_ VCD (middle), and TROPOMI NO_2_ VCD (right) data over Seoul and Gyeonggi (top) and Daegu and Gyeongbuk (bottom). Map created using Google Earth Imagery. Figures generated with Interactive Data Language (IDL) version 8.8.0 (https://www.l3harrisgeospatial.com/Software-Technology/IDL).
